# Pivotal roles for cancer cell–intrinsic mPGES-1 and autocrine EP4 signaling in suppressing antitumor immunity

**DOI:** 10.1172/jci.insight.178644

**Published:** 2024-11-08

**Authors:** Nune Markosyan, Il-Kyu Kim, Charu Arora, Liz Quinones-Ware, Nikhil Joshi, Noah Cheng, Emma Y. Schechter, John W. Tobias, Joseph E. Hochberg, Emily Corse, Kang Liu, Varenka Rodriguez DiBlasi, Li-Chuan (Eric) Chan, Emer M. Smyth, Garret A. FitzGerald, Ben Z. Stanger, Robert H. Vonderheide

**Affiliations:** 1 Abramson Cancer Center, Perelman School of Medicine,; 2 Abramson Family Cancer Research Institute, Department of Medicine, and; 3 Penn Genomics and Sequencing Core, University of Pennsylvania, Philadelphia, Pennsylvania, USA.; 4 Boehringer Ingelheim Pharmaceuticals, Inc., Ridgefield, Connecticut, USA.; 5 Herbert Irving Comprehensive Cancer Center, Columbia University, New York, New York, USA.; 6 Department of Medicine,; 7 Institute of Translational Medicine and Therapeutics, and; 8 Parker Institute for Cancer Immunotherapy, Perelman School of Medicine, University of Pennsylvania, Philadelphia, Pennsylvania, USA.

**Keywords:** Immunology, Oncology, Cancer immunotherapy, Eicosanoids, Mouse models

## Abstract

Tumor cell–derived prostaglandin E_2_ (PGE_2_) is a tumor cell–intrinsic factor that supports immunosuppression in the tumor microenvironment (TME) by acting on the immune cells, but the impact of PGE_2_ signaling in tumor cells on the immunosuppressive TME is unclear. We demonstrate that deleting the PGE_2_ synthesis enzyme or disrupting autocrine PGE_2_ signaling through EP4 receptors on tumor cells reverses the T cell–low, myeloid cell–rich TME, activates T cells, and suppresses tumor growth. Knockout (KO) of *Ptges* (the gene encoding the PGE_2_ synthesis enzyme mPGES-1) or the EP4 receptor gene (*Ptger4*) in KPCY (*Kras^G12D^* *P53^R172H^* *Yfp* *Cre^Pdx^*) pancreatic tumor cells abolished growth of implanted tumors in a T cell–dependent manner. Blockade of the EP4 receptor in combination with immunotherapy, but not immunotherapy alone, induced complete tumor regressions and immunological memory. Mechanistically, *Ptges*- and *Ptger4*-KO tumor cells exhibited altered T and myeloid cell attractant chemokines, became more susceptible to TNF-α–induced killing, and exhibited reduced adenosine synthesis. In hosts treated with an adenosine deaminase inhibitor, *Ptger4*-KO tumor cells accumulated adenosine and gave rise to tumors. These studies reveal an unexpected finding — a nonredundant role for the autocrine mPGES-1/PGE_2_/EP4 signaling axis in pancreatic cancer cells, further nominating mPGES-1 inhibition and EP4 blockade as immune-sensitizing therapy in cancer.

## Abstract 2

The study elucidates a new, unexpected role for mPGES-1 derived PGE2 through EP4 signaling in tumor cells which confers on them a pivotal role in orchestrating the suppression of anti-tumor immunity.

## Introduction

Pancreatic ductal adenocarcinoma (PDAC) continues to carry a dismal prognosis (1), largely due to its resistance to chemotherapy, targeted therapy, and immunotherapy. Checkpoint inhibition with antibodies against PD-1, with or without anti–CTLA-4 antibodies, is rarely effective, related to the immunosuppressive microenvironment in PDAC tumors (2–5). Recent data from our group and others indicate that tumor cell–intrinsic factors in PDAC, such as CXCL1, EPHA2, USP22, EGFR, and the prostaglandin synthesis enzyme COX-2, promote myeloid cell infiltration that negatively impacts T cell infiltration, function, and tumor rejection (6–9).

Prostaglandin E_2_ (PGE_2_) is a potent inflammatory lipid mediator with pronounced immunosuppressive capabilities (10). In pathological conditions, PGE_2_ is mostly produced by reactions catalyzed by cyclooxygenase 2 (COX-2) and microsomal prostaglandin E synthase 1 (mPGES-1) enzymes (11). Many cell types — including tumor cells, endothelial cells, fibroblasts, macrophages, and myeloid-derived suppressor cells (MDSCs) — express COX-2 and mPGES-1, both of which can be induced by growth factors, cytokines, and hypoxia to promote the synthesis and release of PGE_2_ (11). Here, we focused on understanding the protumorigenic mechanism of PGE_2_, given the unusually high expression of COX-2 and mPGES-1 in PDAC tumor cells and the association of their overexpression with poor tumor-free and overall survival in PDAC patients (12). PGE_2_ promotes tumor growth by sustaining chronic inflammation, inducing neoangiogenesis (13), suppressing antitumor immune responses (10, 14–17), and facilitating metastatic dissemination (18). Previously, we have reported evidence for an immunosuppressive role of the PGE_2_ synthesis enzyme COX-2 in breast and pancreatic cancers (7, 19–21). COX-2–selective inhibitors are effective pharmacologic agents, but their use in cancer patients who are predisposed to thrombotic complications is problematic, as these drugs are associated with an enhanced risk of adverse cardiovascular events (22–24). While loss of upstream COX-2 is associated with adverse systemic effects, loss of *Ptges*, the gene encoding the mPGES-1 enzyme, appears to circumvent these complications, as its loss does not appear to affect thrombogenesis or blood pressure (25). We, therefore, hypothesized that targeting this downstream component of the PGE_2_ pathway may provide equal or greater benefits without the risks associated with COX-2 inhibition.

PGE_2_ signals through 4 G protein–coupled receptors, EP1–EP4, that vary in their tissue distribution, ligand affinity, and signaling (26). EP4 is widely distributed, has a high affinity for PGE_2_, activates MAPK and PI3K, drives tumor stem cell features, and induces angiogenesis, lymphangiogenesis, and metastasis in breast cancer and melanoma (26–28). Other data implicate PGE_2_ and EP2/EP4 receptors in a range of immunosuppressive phenotypes in tumors through signaling in NK cells, dendritic cells, MDSCs, and macrophages (15, 17, 29–35). Recent data demonstrate that PGE_2_ limits the expansion of tumor-infiltrating T cells by acting through EP2/EP4 receptors (36, 37). While all these studies demonstrate the immunosuppressive effect of PGE_2_ signaling on immune cells, the role of PGE_2_ signaling in tumor cells themselves has not been evaluated. Here, we deleted the gene encoding mPGES-1 or EP4 in tumor cells to examine the cancer cell–intrinsic roles of these proteins in tumor growth and the tumor immune microenvironment.

## Results

### Ptges is required in cancer cells for pancreatic tumor growth in vivo.

To explore whether tumor intrinsic mPGES-1 is associated with an immunosuppressive tumor microenvironment (TME) in PDAC, we analyzed the expression of *Ptges*, the gene encoding the mPGES-1 enzyme, in clonal tumor cell lines generated from tumors that arose spontaneously in C57BL/6J congenic *Kras^G12D^* *P53^R172H^* *Cre^Pdx^* *Yfp* (KPCY) and KPC mice. These cell lines can be segregated into T cell–low (TL), and T cell–high (TH) groups based on T cell infiltrates of implanted tumors (6). RNA-seq analysis of these cell lines revealed increased transcript counts for *Ptges* in most TL cell lines (Supplemental Figure 1A; supplemental material available online with this article; https://doi.org/10.1172/jci.insight.178644DS1). By quantitative PCR (Q-PCR), all tested TL cell lines had detectable *Ptges* mRNA, whereas only 1 out of 3 TH cell lines had measurable *Ptges* mRNA (Supplemental Figure 1B).

**Figure 1:**
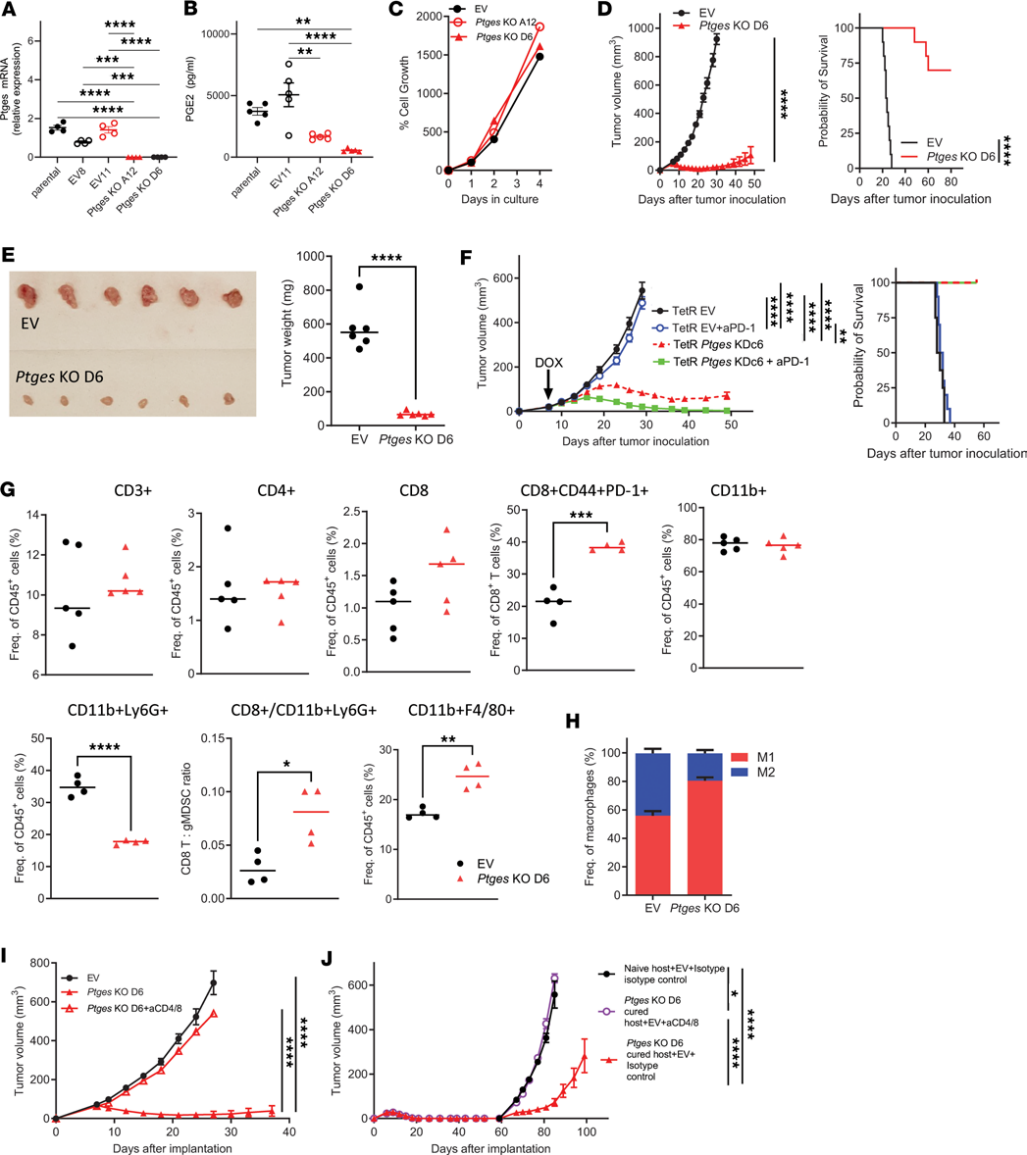
*Ptges* deficiency suppresses tumor growth in a T cell–dependent manner. (**A**) *Ptges* mRNA expression by Q-PCR in parental (non-transduced 6419c5), control (empty vector transduced [EV]), and *Ptges*-KO clonal tumor cell lines A12 and D6 (*n* = 3–4). (**B**) Extracellular PGE_2_ levels measured by ELISA in parental, EV, and *Ptges*-KO clones (*n* = 5). (**C**) EV and *Ptges*-KO clonal cell line growth in vitro (*n* = 2). (**D**) Subcutaneously (s.c.) implanted EV and *Ptges*-KO D6 clone growth in vivo (left, *n* = 10/group) and host survival (right, *n* = 7–13). One out of 3 experiments with similar results shown. (**E**) Orthotopically injected EV and *Ptges*-KO D6 tumors harvested, photographed, and weighed on day 18 after transplantation (*n* = 6). (**F**) Control TetR EV and TetR *Ptges* KDc6 tumor growth in vivo (left) and host survival (right) with and without anti–PD-1 (αPD-1) treatment (*n* = 8–10). (**G**) Flow cytometric analysis of s.c. implanted EV and *Ptges*-KO D6 tumors on day 7 after implantation (*n* = 4–5; 1 of 3 experiments with similar results shown). (**H**) Proportions of M1 (F4/80^+^CD206^–^MHCII^hi^) and M2 (F4/80^+^CD206^+^MHCII^med^) macrophages as a percentage of total macrophages in s.c. implanted EV and *Ptges*-KO D6 tumors (flow cytometry, day 7 after implantation, *n* = 4; 1 of 2 experiments with similar results shown). (**I**) Growth curves of EV and *Ptges*-KO D6 s.c. implanted tumors in hosts receiving CD4^+^ and CD8^+^ cell–depleting or isotype control antibodies (*n* = 5). (**J**) Growth curves of EV tumor cells implanted s.c. into naive hosts receiving isotype control antibodies and hosts that had previously cleared the *Ptges*-KO D6 tumors receiving either CD4^+^ and CD8^+^ T cell–depleting or isotype control antibodies (*n* = 5–8). Data are presented as mean ± SEM (**A**, **B**, **D** [left], **F** [left], **I**, and **J**), mean (**C**), or median (**E** and **G**). Significance was assessed by ordinary 1-way ANOVA with Tukey’s multiple-comparison test (**A** and **B**), 2-way ANOVA with main-effects analysis and Tukey’s multiple-comparison test (**C**), 2-way ANOVA with mix-effects analysis (**D**, left and **F**, left), log-rank Mantel-Cox test (**D**, right), 2-tailed unpaired Student’s *t* test (**E** and **G**), or 2-way ANOVA with mixed-effects analysis and Tukey’s multiple-comparison test (**I** and **J**). For all figures, *P* < 0.05 was considered statistically significant. *P < 0.05; **P < 0.01; ***P < 0.001; ****P < 0.0001.

The TL cell line 6419c5 was then transduced using lentiviral particles containing CRISPR/Cas9 with either no guide RNA — empty vector (EV), or single guide RNA (sgRNA) against mouse *Ptges*. Complete knockout (KO) of *Ptges* was confirmed by Q-PCR in clones A12 and D6 (Figure 1A). PGE_2_ levels in culture media from parental and control EV cells were similar, whereas *Ptges*-KO A12 and D6 produced significantly lower, but detectable, levels of PGE_2_ (Figure 1B) since other PGE_2_ producing enzymes, COX-1, mPGES-2, and cPGES were not targeted. Although enzyme ablation did not affect the in vitro growth of KO cell lines (Figure 1C), in vivo growth following subcutaneous (s.c.) implantation was severely affected*,* resulting in tumor-free survival of most host mice (Figure 1D). Similarly, orthotopically implanted *Ptges*-KO tumors were significantly smaller than control EV tumors, on day 18 after implantation (Figure 1E). Thus, *Ptges* loss and the decreased amount of tumor cell–released PGE_2_ profoundly affects PDAC tumor growth in vivo but not in vitro.

To further confirm the role of *Ptges* in tumor growth and to exclude a possible role of Cas9 antigenicity causing suppressed tumor growth, we knocked down (KD) *Ptges* in the same 6419c5 TL PDAC cell line through doxycycline-inducible (DOX-inducible) shRNA (TetR *Ptges* KD). To account for a possible plasmid effect, the control cell line was transduced with the same backbone vector with no shRNA (TetR EV). *Ptges* KD was verified by Q-PCR in various single-cell clones generated from TetR *Ptges* KD (Supplemental Figure 1C). The control TetR EV and TetR *Ptges* KDc6 clones were implanted into WT mice and DOX treatment of both groups was initiated 7 days after implantation. DOX-inducible KD and the timing of the induction mimic pharmacological inhibition of mPGES-1 only in tumor cells at the early stage of the disease. The in vivo KD of *Ptges* was confirmed by measuring decreased PGE_2_ levels in the urine of TetR *Ptges* KDc6 tumor–bearing mice compared with the control TetR EV tumor–bearing hosts (Supplemental Figure 1D). The growth of the TetR *Ptges* KDc6 tumors was greatly suppressed when DOX administration was started on day 7 after implantation (Figure 1F), but the effect was attenuated when *Ptges* KD was initiated 14 days after implantation (Supplemental Figure 1E). When treated with anti–PD-1 (αPD-1), 5 out of the 8 TetR *Ptges* KDc6 tumor–bearing mice in the day 7 induction group achieved complete tumor regression (Figure 1F). *Ptges* KD resulted in 100% survival of host mice 60 days after transplantation (Figure 1F), confirming the pivotal role of this enzyme in PDAC tumor progression.

To explore the potential role of mPGES-1/*Ptges* in tumor types beyond pancreatic cancer, we examined the effects of global or epithelial cell–specific loss of *Ptges* in the *Her2/neu* model of mammary tumorigenesis. We measured tumor multiplicity at 22 weeks of age in *Her2/neu* transgenic mice with a germline deletion of one (*Ptges^+/–^*) or both (*Ptges^–/–^*) copies of the *Ptges* gene or in mice lacking both copies of *Ptges* in mammary epithelial cells only (*Ptges*^epi^). All gene deletions, including the epithelial cell–specific *Ptges* KO, led to reductions in tumor burden (Supplemental Figure 2, A and B), indicating that tumor cell–intrinsic mPGES-1 plays a role in carcinogenesis of the mammary gland, as it does in the pancreas.

Given the known effects of prostanoids on immune cells, we next evaluated immune infiltration into s.c. tumors 7 days after implantation, when *Ptges*-KO tumors were at their maximal size. By flow cytometry, *Ptges*-KO tumors trended toward increased proportions of activated CD44^+^PD-1^+^CD8^+^ cytotoxic T cells (CTLs) and lower proportions of CD11b^+^Ly6G^+^ myeloid cells (Figure 1G) that we had previously shown to be immunosuppressive granulocytic MDSCs (gMDSCs; refs. 38, 39). As a result, the ratio of CTLs to gMDSCs was significantly higher in *Ptges*-KO tumors (Figure 1G). Immunohistochemical analysis showed that in contrast to control (EV) tumors, where T cells were largely confined to the periphery, *Ptges*-KO tumors had more tumor-infiltrating T cells and fewer Ly6G^+^ cells (Supplemental Figure 3). *Ptges*-KO tumors also had larger relative population of CD11b^+^F4/80^+^ macrophages (Figure 1G) and M1 type CD206^–^MHCII^hi^ macrophages (Figure 1H). Knocking out *Ptges* in another TL PDAC clonal cell line, 6694c2 (6), had similar effects on the TME (Supplemental Figure 4, A and B). Thus, *Ptges* loss in pancreatic cancer cells results in a substantial reshaping of the immune TME to a less immunosuppressive makeup.

To test whether these immune changes functionally contribute to the in vivo growth phenotype of *Ptges*-KO tumors, immunocompetent host mice were depleted of T cells before implantation and throughout the tumor growth period (Supplemental Figure 5). Deletion of CD4^+^ and CD8^+^ T cells resulted in a complete rescue of tumor growth (Figure 1I), indicating that T cells are necessary for tumor clearance in the absence of *Ptges*. Next, we asked whether this immune antitumor effect is associated with the development of immunological memory and challenged the mice that had cleared *Ptges*-KO tumors (“cure”) by reinjecting control *Ptges* intact, EV tumor cells either with or without T cell depletion. EV tumors implanted into cured mice showed a significant delay in tumor growth, an effect that was abolished by T cell depletion (Figure 1J). These results suggest that the clearance of *Ptges*-KO tumor cells is T cell dependent and results in immunological memory.

### PGE_2_ receptor EP4 in cancer cells has a pivotal role in the growth of pancreatic and mammary tumors.

*Ptges*-derived PGE_2_ mediates immunosuppression by acting through EP2 and EP4 receptors on various immune cells (29–32, 40). However, EP4 is also expressed on most epithelial and tumor cells (26, 27, 41), raising the prospect that PGE_2_ may also exert direct, autocrine effects on tumor cells. In addition to TL 6419c5 and TH 2838c3 PDAC cell lines (6), we checked 4662 MD7 and 4662 MD10 PDAC tumor cell lines to assess the degree of T cell infiltration and EP2 and EP4 receptor expression. Both 4662 clonal cell lines showed intermediate T cell infiltration (TI) (Supplemental Figure 6A). By Q-PCR, EP2 mRNA (encoded by *Ptger2*) was mostly undetectable in all cell lines, whereas EP4 (encoded by *Ptger4*) was present in all cell lines, with intermediate expression in TI 4662 and significantly higher expression in TL 6419c5 cell lines (Supplemental Figure 6B).

**Figure 2:**
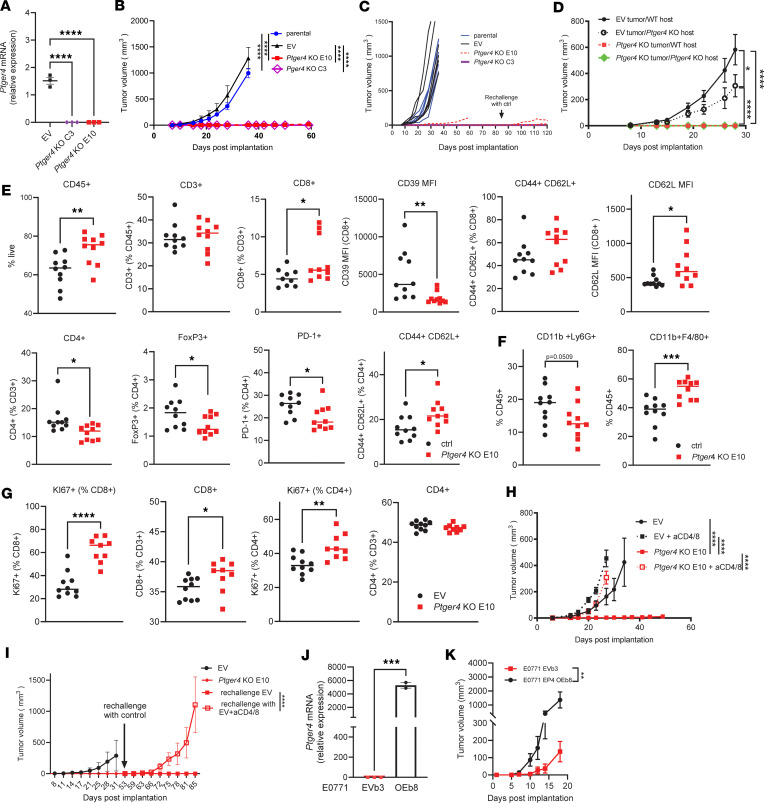
*Ptger4* KO in T cell–low tumor cell line abolishes implanted tumor growth in a T cell–dependent manner. (**A**) *Ptger4* mRNA expression by Q-PCR in control EV and TL 6419c5 *Ptger4*-KO clonal tumor cell lines (*n* = 3). (**B**) S.c. implanted non-transduced 6419c5 (parental), EV, and *Ptger4*-KO tumor growth (*n* = 6, 1 of 3 experiments with similar results shown). (**C**) Individual tumor growth curves of tumors in **B**. The black vertical arrow indicates the rechallenge of tumor-free mice with control cell implants on day 85 (*n* = 11: 5 and 6 mice cured of *Ptger4*-KO E10 and *Ptger4*-KO C3 tumors, respectively). (**D**) Control and *Ptger4*-KO tumor growth in WT and *Ptger4*-KO hosts (*n* = 10, 1 of 2 experiments with similar results shown). (**E**) Flow cytometric analysis of T cells in control and *Ptger4*-KO s.c. tumors 11 days after implantation (*n* = 10, 1 of 3 experiments with similar results shown). (**F**) Flow cytometric analysis of myeloid cells in control and *Ptger4*-KO s.c. tumors, 11 days after implantation (*n* = 10, 1 of 3 experiments with similar results shown). (**G**) Flow cytometric analysis of s.c. tumor-draining lymph nodes 10 days after implantation (*n* = 9–10, 1 of 3 experiments with similar results shown). (**H**) S.c. implanted control and *Ptger4*-KO tumor growth with and without CD4^+^ and CD8^+^ T cell depletion (*n* = 10). (**I**) Rechallenge control tumor growth with and without CD4^+^ and CD8^+^ T cell depletion in hosts after complete regression of *Ptger4*-KO s.c. implants (*n* = 10). (**J**) *Ptger4* mRNA expression by Q-PCR in control E0771 EVb3 and E0771 OEb8 mammary clonal tumor cell lines (*n* = 3). (**K**) Growth curves of orthotopically implanted E0771 EVb3 and E0771 OEb8 mammary tumor cell lines (*n* = 4–5). Data are presented as median (**A**, **E**, and **F**), mean ± SEM (**B**, **D**, and **H**–**J**), and in **C**, each line represents an individual tumor. Significance was assessed by ordinary 1-way ANOVA with Tukey’s multiple-comparison test (**A**), 2-way ANOVA with Tukey’s multiple-comparison test for main-effects analysis (**B**, **D**, **H**, **I**, and **K**), or 2-tailed unpaired Student’s *t* test (**E**–**G** and **J**). *P < 0.05; **P < 0.01; ***P < 0.001; ****P < 0.0001.

To assess the impact of EP4 loss in epithelial cells, we used CRISPR/Cas9 to knock out *Ptger4*, the gene encoding EP4, in the 6419c5 TL PDAC clonal cell line (*Ptger4* KO). We confirmed *Ptger4* KO by Q-PCR in *Ptger4*-KO E10 and C3 clones (Figure 2A). In addition, intracellular cAMP measurements demonstrated that *Ptger4*-KO E10 cells fail to upregulate cAMP production in response to PGE_2_ treatment in vitro (Supplemental Figure 6C). Both KO lines (C3 and E10) grew well in vitro, although the growth of C3 was significantly slower than that of EV control cells (Supplemental Figure 6D). By contrast, neither KO clone formed tumors in vivo following s.c. implantation, while non-modified parental 6419c5 and EV cells grew at similar rates (Figure 2B). When mice cured of their *Ptger4*-KO tumors were challenged with control 6419c5 EV cells, all but 1 mouse rejected the tumors (Figure 2C). Growth of *Ptger4*-KO cells was also markedly impeded in the orthotopic setting (Supplemental Figure 6E, left). In contrast with the near-universal rejection of s.c. implanted *Ptger4*-KO tumors, only 3 out of 10 hosts were able to clear orthotopically implanted KO tumors (Supplemental Figure 6E, middle and right), indicating that the anatomical niche affects the antitumor response (42).

Given the unexpected robust antitumor effect of *Ptger4* deletion in tumor cells, we sought to determine the relative contribution of tumor cell–intrinsic versus –extrinsic EP4 signaling in tumor growth. To this end, we employed tamoxifen-inducible *Ptger4*-KO mice (Supplemental Figure 7A) as hosts for s.c. implantation of control EV or *Ptger4*-KO tumor cells. As expected, *Ptger4*-KO cells failed to grow in either WT or *Ptger4*-KO hosts (Figure 2D). By contrast, control cells with intact *Ptger4* were able to grow in *Ptger4*-KO hosts, albeit at a significantly slower rate compared with control hosts (Figure 2D). These results suggest that EP4 signaling in tumor cells, more than signaling in other components of the TME, drives PGE_2_-mediated immunosuppression in pancreatic tumors.

To further investigate the possible effects of tumor cell–intrinsic EP4 signaling on the TME, flow cytometric analysis of s.c. implanted tumors was performed 11 days after implantation. *Ptger4*-KO tumors had a higher proportion of tumor-infiltrating CD45^+^ immune and CD8^+^ T cells compared with the control tumors (Figure 2E). In addition, CD8^+^ cells had lower expression of the T cell exhaustion marker CD39, but a higher proportion of CD44^+^CD62L^+^ central memory cells. CD4^+^ T cells in *Ptger4*-KO tumors had fewer FoxP3^+^ and PD-1^+^ and more CD62L^+^ cells (Figure 2E), indicating a decrease in regulatory and an increase in memory T helper cells. Moreover, *Ptger4*-KO implants had decreased gMDSC and increased macrophage populations compared with the controls (Figure 2F). In addition to the changes in TME, the analysis of tumor-draining lymph nodes revealed increased proportions of Ki67^+^ proliferative CD8^+^ and CD4^+^ T cells in *Ptger4*-KO tumor–bearing mice (Figure 2G), suggesting that the disruption of tumor cell–intrinsic EP4 signaling may have systemic effects. Next, we tested the role of T cells in the rejection of *Ptger4*-KO tumors by depleting host mice of CD4^+^ and CD8^+^ T cells. As with *Ptges*-KO tumor cells, the rejection of primary *Ptger4*-KO cells as well as rechallenged control EV tumor cells in cured mice was T cell dependent (Figure 2, H and I). Thus, we conclude that tumor cells are dependent on EP4, a receptor for PGE_2_, for growth in immunocompetent hosts.

Next, we sought to further test the role of tumor cell EP4 signaling by overexpressing it on a mammary tumor cell line with no endogenous *Ptger4* expression. Two mammary tumor cell lines, PY8119 and E0771, were similar in terms of the absence of *Ptger2* expression but showed quite different levels of *Ptger4*, with E0771 being almost completely deprived of the receptor expression (Supplemental Figure 7B). We transduced the E0771 tumor cell line to overexpress *Ptger4* (E0771 OE). To account for any plasmid effects, the control cells were transduced with the empty vector (E0771 EV). After generating single-cell clones and confirming *Ptger4* overexpression by Q-PCR (Supplemental Figure 7C and Figure 2J), E0771 EVb3 and E0771 OEb8 clones were selected for further experiments. The control and *Ptger4*-overexpressing mammary tumor cells grew at the same rate in vitro (Supplemental Figure 7D). When implanted orthotopically into the mammary fat pads of syngeneic C57BL/6J mice, as early as 9 days after implantation, E0771 OEb8 tumors were significantly larger than the controls (Supplemental Figure 7E), reaching a 10-fold difference by day 18 (Figure 2K). The TME analysis on day 9 after implantation demonstrated that *Ptger4*-overexpressing mammary tumors had significantly reduced counts of immune cells compared with the controls (Supplemental Figure 7F), confirming the role of tumor cell–intrinsic EP4 signaling in suppressing antitumor immunity and promoting tumor growth.

### Disruption of PGE_2_ signaling in tumor cells exerts antitumor activity through altered production of cytokines, chemokines, and immunosuppressive adenosine.

**Figure 3:**
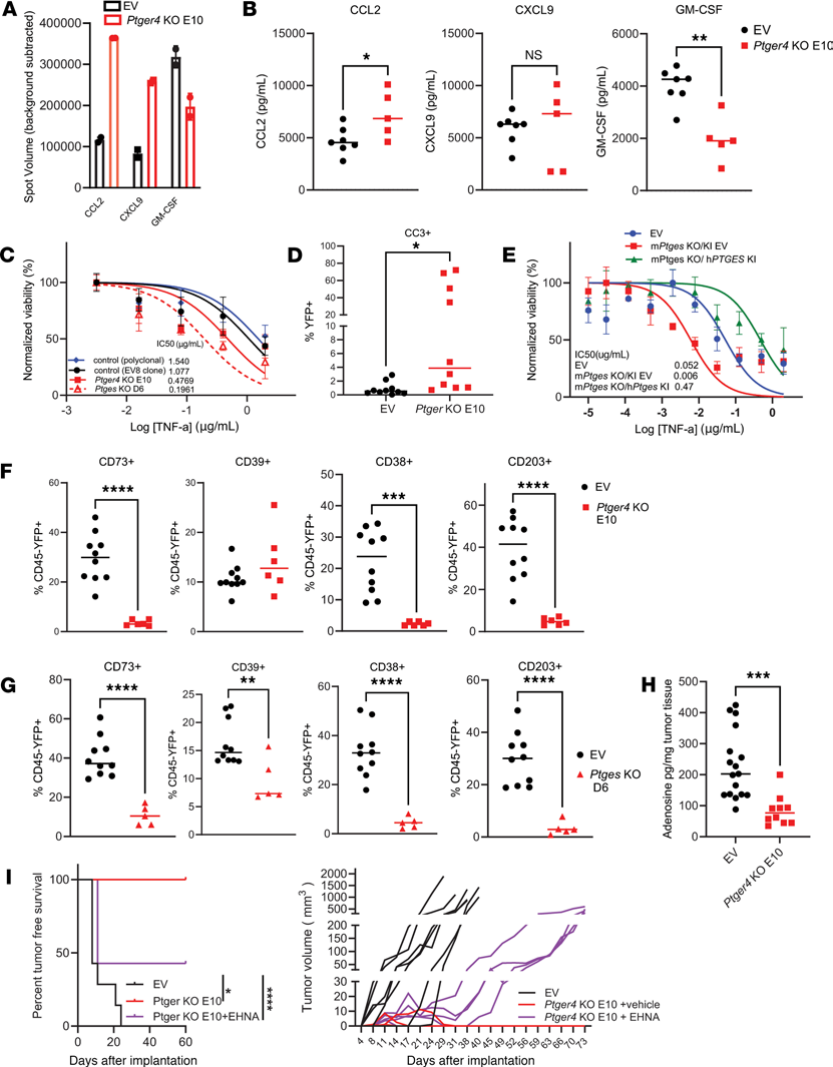
Disruption of PGE_2_ signaling in tumor cells changes the TME through altered production of cytokines, chemokines, and immunosuppressive adenosine. (**A**) Cytokine and chemokine array analysis of media from control (7 pooled samples) and *Ptger4*-KO (8 pooled samples) tumors cultured ex vivo in hypoxic conditions (average of 2 technical replicates shown). (**B**) Cytokines from **A** measured using multiplex bead-based assay in the media of control and *Ptger4*-KO tumors cultured ex vivo in hypoxic conditions (*n* = 5–7; in the control group, all data points represent individual tumors and in *Ptger4*-KO group, 3 data points represent individual tumors and 2 data points represent 2 pooled samples each). (**C**) Cell viability in vitro assay after 48-hour incubation of the indicated cell lines with indicated concentrations of TNF-α. Cell viability in treatment groups is shown as a percentage of untreated control cells (*n* = 3/cell line/TNF-α concentration). (**D**) Proportion of cleaved caspase 3^+^ (CC3^+^) tumor cells in *Ptger4*-KO and control tumors by flow cytometry 11 days after implantation (*n* = 10). (**E**) Cell viability in vitro assay after 48-hour incubation of the indicated cell lines with indicated concentrations of TNF-α. Cell viability in treatment groups is shown as a percentage of untreated control cells (*n* = 3/cell line/TNF-α concentration). (**F**) Flow cytometric analysis of adenosine synthesis pathway enzymes in EV and *Ptger4*-KO tumors, 11 days after s.c. implantation (*n* = 6–10). (**G**) Flow cytometric analysis of adenosine synthesis pathway enzymes in EV and *Ptges*-KO tumors, 10 days after s.c. implantation (*n* = 6–10). (**H**) Adenosine levels per mg tumor tissue measured by mass spectrometry in media of control EV and *Ptger4*-KO tumors cultured ex vivo for 24 hours under hypoxic conditions in the presence of 1 μM PGE2 and 10 μM EHNA (adenosine deaminase inhibitor). Combined data from 2 separate experiments shown (*n* = 11–17). (**I**) Survival (left) and individual growth curves (right) of s.c. control EV and *Ptger4*-KO tumors implanted in hosts receiving either vehicle or 0.3 mg/ml EHNA every other day (*n* = 7). Data are presented as mean ± SEM (**A**), median (**B**, **D**, and **F**–**H**), and in **I** (right), each line represents an individual tumor. Significance was assessed by 2-tailed unpaired Student’s *t* test (**B**, **D**, **F**–**H**), global nonlinear regression analysis in GraphPad Prism (**C** and **E**), or log-rank Mantel-Cox test (**I**, left). *P < 0.05; **P < 0.01; ***P < 0.001; ****P < 0.0001.

To check the changes in cytokine and chemokine profiles caused by EP4 deletion in tumor cells, *Ptger4*-KO and control tumors were harvested 10 days after implantation and cultured ex vivo for 24 hours for conditioned media analysis. By cytokine array and bead assay analyses, compared with control tumors, *Ptger4*-KO tumors exhibited an increase in the levels of CCL2 (monocyte chemoattractant) and a decrease in levels of GM-CSF (myeloid chemoattractant), and a trend toward increased T cell recruitment factor CXCL9 (Figure 3, A and B). These changes in tumor cytokine profile are consistent with increased macrophages and T cells and decreased gMDSC populations observed in *Ptger4*-KO tumors.

To identify other genes and pathways affected by the disruption of PGE_2_/EP4 signaling, we performed RNA-seq on in vitro–cultured and in vivo–sorted tumor cells and bulk tumors from control, *Ptges*-KO, and *Ptger4*-KO cell lines. Gene set enrichment analysis (GSEA) revealed that TNF-α signaling via NF-κB was the top enriched Hallmark pathway in both KO cell lines in vitro (not shown), in vivo, and in bulk tumors (Supplemental Figure 8A). Consistent with this finding, transcript counts for TNF-α receptors *Tnfrsf1a* and *Tnfrsf1b* were significantly higher in KO tumors (Supplemental Figure 8B). When tested in vitro, *Ptges*-KO and *Ptger4*-KO tumor cells were 2- to 5-fold more susceptible to TNF-α–induced killing compared with parental and control EV cell lines (Figure 3C). Accordingly, *Ptger4*-KO tumors had a higher proportion of dying, cleaved caspase 3^+^ (CC3^+^) cells, 10 days after implantation (Figure 3D). To investigate whether restored PGE_2_ production and signaling by tumor cells will reverse their resistance to TNF-α–induced killing, we knocked in (KI) the human *PTGES* gene (h*PTGES*) into the mouse *Ptges*-KO D6 clone, denoting these cells as m*Ptges*-KO/h*PTGES*-KI tumor cells. Additionally, we generated m*Ptges*-KO cells transduced with the empty KI vector (m*Ptges* KO/KI EV). We confirmed m*Ptges* expression in control 6419c5 cell line (EV), but not in m*Ptges*-KO/KI EV or m*Ptges*-KO/h*PTGES*-KI cells by Q-PCR (Supplemental Figure 8C, left). As expected, the h*PTGES* gene was detectable only in m*Ptges*-KO/h*PTGES*-KI cells (Supplemental Figure 8C, right). Consequently, the control EV and m*Ptges*-KO/h*PTGES*-KI cells released PGE_2_ in vitro, but the EV cell culture media had almost non-measurable levels of PGE_2_ (Supplemental Figure 8D). In vitro TNF-α killing assays revealed that m*Ptges*-KO/KI EV cells that were deprived of PGE_2_ were approximately 9-fold more sensitive to TNF-α–induced killing, while overexpression of h*PTGES* resulted in approximately 9-fold increased resistance of m*Ptges*-KO/h*PTGES*-KI cells compared with the control (Figure 3E). These results suggest that in the absence of tumor cell–derived PGE_2_ or autocrine EP4 signaling, PDAC cells are more prone to TNF-α–induced killing.

RNA-seq analysis of bulk *Ptger4*-KO tumors (but not in vitro– and in vivo–sorted KO tumor cells) indicated significantly decreased TGF-β signaling compared with the controls (Supplemental Figure 9A). TGF-β signals through phosphorylated-SMAD (p-SMAD) transcription factors (43). By Western blot analysis, at baseline, cultured tumor cells had low levels of p-SMAD3, which was upregulated by TGF-β and PGE_2_ incubation (Supplemental Figure 9B). Even though both control and *Ptger4*-KO tumor cells showed an increase in p-SMAD3 protein upon TGF-β and PGE_2_ exposure, EP4-deficient tumor cells had lower levels of p-SMAD3 compared with the controls, with and without treatment, indicating a deficiency in TGF-β signaling (Supplemental Figure 9B).

Previous reports have demonstrated that the adenosine pathway drives the immunosuppressive TME in PDAC (44) and is upregulated by TGF-β signaling through SMAD transcription factors and hypoxia (45). Indeed, we also found that *Nt5E* and *ADORA2B*, genes encoding the adenosine-synthesizing enzyme CD73 and adenosine receptor A2BR, respectively, correlate with poor PDAC patient survival (Supplemental Figure 9C). RNA-seq analysis of YFP^+^ tumor cells sorted from s.c. implanted tumors revealed a decrease in transcript counts for genes encoding 3 adenosine-synthesizing enzymes, CD73, CD38, and CD203a, and an increase in CD39 transcript counts, in *Ptges*-KO and *Ptger4*-KO tumors (Supplemental Figure 9D). *Dpp4*, the gene encoding the CD26 receptor that is necessary for adenosine deaminase activity (46), was upregulated in *Ptger4*-KO and *Ptges*-KO tumor cells (Supplemental Figure 9D). Flow cytometric analysis 10 days after implantation confirmed the decrease in adenosine-synthesizing enzymes CD73, CD38, and CD203 in tumor cells from both KO lines, while CD39 protein was unchanged in *Ptger4*-KO and decreased in *Ptges*-KO tumors (Figure 3, F and G). These results are consistent with the hypothesis that disruption of mPGES-1/PGE_2_/EP4 signaling in tumor cells inhibits their ability to synthesize immunosuppressive adenosine.

To further evaluate this hypothesis, we measured released adenosine levels ex vivo in conditioned media of tumors harvested 10 days after implantation and cultured for 24 hours under hypoxic conditions in the presence of PGE_2_ and EHNA, a potent adenosine deaminase inhibitor that allows for adenosine accumulation in tissues (47). *Ptger4*-KO tumors produced significantly less adenosine per unit of tumor weight (Figure 3H). To test whether elevating adenosine levels in *Ptger*-KO tumors could prevent their rejection, *Ptger4*-KO tumor–bearing mice were randomized into the vehicle and EHNA treatment groups. Consistently, all 7 mice in the untreated group rejected the KO tumors; by contrast, in 4 out of 7 mice treated with EHNA, *Ptger4*-KO E10 tumor cells formed tumors by day 11 after implantation that continued growing, albeit with slower kinetics than control tumors (Figure 3I). Together, these results suggest that the mPGES-1/PGE_2_/EP4 axis supports tumor progression at least in part by regulating adenosine production by tumor cells.

### Pharmacological inhibition of PGE_2_ signaling sensitizes tumors to immunotherapy and chemotherapy.

**Figure 4:**
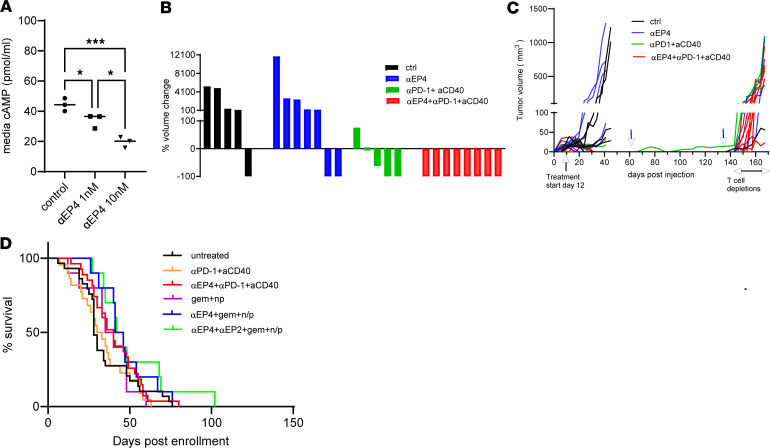
Pharmacological inhibition of PGE_2_ signaling sensitizes tumors to immunotherapy and chemotherapy. (**A**) Released cAMP measured by ELISA in culture media of TI 4662 MD7 PDAC clonal cell line treated either with vehicle (control) or 1 nM and 10 nM EP4 antagonist ONO-AE3-208 (αEP4) for 48 hours (*n* = 3). (**B**) S.c. implanted 4662 MD7 tumor volume change with and without indicated treatments, 41 days after implantation compared to the volume at the start of the treatment (each bar represents a tumor). (**C**) Individual tumor growth curves of the tumors in **A**, before and after rechallenges. The black vertical arrow indicates treatment start, blue arrows indicate first and second rechallenges on days 62 and 136, and horizontal black arrows indicate the period host mice were depleted of T cells (*n* = 5–8 initial implant, *n* = 12 rechallenge group: 2, 2, and 8 cured mice from αEP4, αPD-1+aCD40, and αEP4+αPD-1+aCD40 groups, respectively). (**D**) Postenrollment survival of KPC and KPCY mice with indicated treatments. Data are presented as median (**A**), individual columns (**B**), and in **C**, each line represents individual tumors. Significance was assessed by ordinary 1-way ANOVA with Tukey’s multiple-comparison test (**A**). *P < 0.05; ***P < 0.001.

Next, we sought to extend these findings in a preclinical setting. Several small molecule inhibitors of mPGES-1 have been reported and were shown to have activity in rodents (48–50); however, the efficacy of these inhibitors may be strain dependent, as they failed to decrease systemic PGE_2_ levels in C57BL/6J mice (Supplemental Figure 10), precluding us from testing their effects on tumors. Instead, we focused on EP4 as a target. Treatment of TI 4662 MD7 tumor cells (Supplemental Figure 6, A and B) in vitro with the EP4 antagonist ONO-AE3-208 (αEP4) resulted in a dose-dependent inhibition of EP4 signaling, as measured by cAMP production (Figure 4A). αEP4 treatment had a small, but significant, effect on in vitro growth (Supplemental Figure 11A), prompting us to test the effects of this compound in vivo. Twelve days after s.c. implantation of 4662 MD7 cells, tumor-bearing animals were treated with either αEP4 or αPD-1 plus agonistic CD40 (aCD40), or the combination. While αEP4 alone had minimal effect on tumor growth and αPD-1+aCD40 immunotherapy resulted in a heterogeneous response, the combination resulted in complete regression of all s.c. implanted tumors (Figure 4B). Another TI clonal tumor cell line, 4662 MD10 (Supplemental Figure 6, A and B), showed a similar response to combination therapy in vivo (Supplemental Figure 11B). Mice that had been cured of 4662 MD7 tumors in Figure 4B were rechallenged with the same cell line 62 days after the first implantation (Figure 4C). Although the cured and rechallenged mice did not receive any of the treatments, all but 1 mouse rejected the rechallenge tumors. Cured mice that had also rejected the rechallenged tumors were then depleted of T cells and received a third tumor implant. In the absence of T cells, all the host mice developed tumors. Similar results were obtained with 4662 MD10 clonal cell line (Supplemental Figure 11C).

Finally, we tested various treatment regimens in tumor-bearing KPC mice. Immunotherapy with αPD-1+aCD40 or chemotherapy with gemcitabine and nab-paclitaxel (np) had no significant effect on survival (Figure 4D). Adding αEP4 to immunotherapy resulted in a trend toward improved survival that was not statistically significant. When compared directly to the untreated group, only 2 treatment regimens, a combination of αEP4 with chemotherapy and αEP4 + αEP2 + chemotherapy increased the survival after the treatment start (*P* = 0.048 and *P* = 0.037 by Gehan-Breslow-Wilcoxen survival test, respectively). Thus, pharmacological inhibition of EP4 has limited efficacy on its own, but sensitizes implanted tumors to immunotherapy and can slow down the growth of autochthonous tumors in combination with chemotherapy.

## Discussion

We have previously reported that tumor cell–intrinsic factors prevent effective antitumor immune responses in PDAC and contribute to resistance to immune therapies (6–9). The PGE_2_ synthesis enzyme COX-2, a target for non-steroidal antiinflammatory drugs including selective inhibitors, emerged as one such factor (7, 21) and has been shown to support an immunosuppressive TME in other cancer types as well (10, 34, 51). Although potentially a therapeutic option, COX-2–selective inhibitors have major limitations due to the risk of catastrophic vascular events and the potential for promoting tumor growth through different mechanisms (52). Here, we focused on a downstream component of the pathway as a logical alternative to COX-2–selective inhibition.

mPGES-1 is associated with poor survival and markers of immunosuppression in human pancreatic cancer, melanoma, and neuroblastoma, as well as in mouse models of neuroblastoma and melanoma (12, 49, 53–55). Utilizing the KPC mouse model of PDAC, we found that *Ptges* deletion is sufficient to suppress or abrogate the growth of s.c. and orthotopically implanted tumors. The effect was primarily T cell dependent, and rechallenge experiments revealed that the rejection of *Ptges*-KO tumors resulted in T cell memory. Growth inhibition was associated with changes in the TME, with increased infiltration of T cells (especially activated CD8^+^ T cells) and M1-like macrophages observed in *Ptges*-deleted tumors. While these TME changes can be attributed to the decrease in PGE_2_ levels and its direct effect on immune cells, it is likely that suppressed adenosine production by *Ptges*-KO tumor cells is a major factor in restoring antitumor immunity. This notion is supported by the finding that *Ptger4*-KO tumors with unaltered PGE_2_ synthesis enzymes, but decreased adenosine production by tumor cells, show a similar degree of tumor growth suppression. The effects of *Ptges* loss in tumor cells extended beyond pancreatic cancer, as tumor cell–intrinsic KO of *Ptges* suppressed tumor growth in a breast cancer model as well. Our findings are in line with studies in melanoma, where *Ptges* deletion resulted in an increase in CD8^+^ T cell infiltration that synergized with αPD-1 therapy (55), and another model of breast cancer, where *Ptges* KD attenuated the extent of tumor-infiltrating monocytic MDSCs (mMDSCs) (40). Overall, these data indicate that *Ptges/*mPGES-1 activity in tumor cells is a pivotal regulator of tumor immunity.

Perhaps the most surprising result in our studies was the finding that the major product of mPGES-1 — the prostanoid PGE_2_ — exerts its immunosuppressive effects mainly through its activity on cancer cells rather than immune cells in the TME. So far, prevailing evidence has pointed to PGE_2_ signaling through EP2 and EP4 in immune cells as mediators of immunosuppressive phenotype (29–32, 36, 37, 56, 57). However, the higher expression of EP4 on TL tumor cells led us to hypothesize that EP4 signaling in tumor cells may have a role in tumor immune regulation. Here, we report that deletion of the *Ptger4* gene specifically in tumor cells was sufficient to abrogate tumor growth in hosts where EP2 and EP4 signaling in immune cells was intact. The rejection of *Ptger4*-KO tumors was T cell dependent and had a vaccine effect, as when rechallenged, the cured host mice were able to reject the EP4-sufficient tumors as well. Tumor cell–intrinsic EP4 appeared to be more important in tumor rejection than the same receptors in stromal and immune cells, as *Ptger4*-KO tumor cells were rejected in both WT and *Ptger4*-KO hosts, whereas control cells grew in *Ptger4*-KO hosts, albeit at a slower rate compared with normal hosts. Moreover, *Ptger4* KO resulted in more robust tumor growth suppression than *Ptges* KO; s.c. implanted *Ptger4*-KO tumors were almost universally rejected, while approximately 30% of *Ptges*-KO tumors escaped antitumor immunity, likely through the residual PGE_2_ signaling through intact EP4 receptors on tumor cells that according to Figure 2D have a non-redundant role in creating immunosuppressive TME.

We found that mPGES-1/PGE_2_/EP4 signaling in tumor cells is protective against antitumor immunity through at least 2 mechanisms: (i) increased intrinsic resistance to the cytotoxic effects of TNF-α, and (ii) creation of an immunosuppressed environment through the production of adenosine. Adenosine is a potent immunosuppressive molecule, and enzymes responsible for its synthesis as well as its cognate receptors A2AR and A2BR are targets for various drugs currently in clinical trials (58, 59). Our findings suggest that autocrine mPGES-1/PGE_2_/EP4 signaling creates an immunosuppressive environment by inducing the synthesis and release of adenosine; in agreement with these findings, application of the adenosine deaminase inhibitor EHNA partially restored the growth of *Ptger4*-KO tumors. Our results are consistent with other reports demonstrating an important role for adenosine signaling in PDAC. The adenosine synthesis enzyme Nt5e/CD73 is one of the top upregulated genes in the epithelial gene signatures for the most immunosuppressed molecular types of mouse and human pancreatic cancer, and small-molecule inhibitors of CD73 can reduce autochthonous and implanted pancreatic tumor development and growth (44). Similarly, tumor cell–derived PGE_2_ induced adenosine production by human and mouse mMDSCs, and treatment with PEGylated adenosine deaminase improved the response to αPD-1 (40). Taken together, these results suggest that tumor cells respond to mPGES-1/PGE_2_/EP4 signaling by synthesizing and releasing adenosine. While our preliminary data implicate tumor cell–derived adenosine, TGF-β, and SMAD3 in mediating tumor cell–intrinsic EP4 signaling induced immunosuppression, the precise mechanisms need further investigation.

In our mouse model, loss of the *Ptgs2* gene encoding COX-2 suppresses tumor growth to some extent (7), and the growth-suppressive effects of deleting *Ptges* or *Ptger4* are far more robust, a finding with important translational implications. EP4 antagonist alone used at a dose shown to be effective in breast cancer (60) had almost no effect in the implanted PDAC model, likely because of the highly desmoplastic stroma and vascular collapse impeding drug perfusion and diffusion (61). However, our data demonstrate that intratumoral levels of EP4 antagonist were sufficient to sensitize tumors to immunotherapy and chemotherapy, supporting the clinical investigation of EP4 antagonists as immune-sensitizing agents. Although we were unable to confirm the activity of small molecules previously reported to inhibit mPGES-1 (48–50), our findings likewise provide a rationale for the clinical evaluation of effective mPGES-1 inhibitors.

In conclusion, the current study demonstrates that the mPGES-1/PGE_2_/EP4 axis is a major immunosuppressive pathway in pancreatic and other cancers. While the direct suppressive effect of tumor-derived PGE_2_ on immune cells has been recognized in various tumor types, our findings demonstrate, we believe for the first time, that PGE_2_ has a direct effect on tumor cells that activates non-redundant immunosuppressive mechanisms sufficient to reject the tumors. Hence, inhibition of mPGES-1 or EP4 signaling might be a strong clinical alternative to COX-2 inhibition in cancer treatment.

## Methods

### Sex as a biological variable.

For s.c. and orthotopic tumor implantation, 6- to 8-week-old female C57BL/6J WT mice were used. At this younger age, sex-based differences in the immune response are expected to be absent or minimal. Fourteen- to 22-week-old male and female *Ptger4*-KO mice were enrolled in the Figure 2D study. Seven- to 28-week-old male and female autochthonous KPC tumor–bearing mice were enrolled in the Figure 4D study.

### Animals.

*Ptges^fl/fl^* and *Ptges* global KO (*Ptges^–/–^*) C57BL/6J mice (62, 63) were backcrossed for 9 generations to the FVB background. The full backcrossing was confirmed by microsatellite marker analysis (IDEXX BioResearch). *Ptges^fl/fl^* mice on the FVB background are denoted as control. For mammary epithelial cell *Ptges* deletion, the control (*Ptges^fl/fl^*) mice were crossed with FVB mice expressing Cre recombinase under the control of the mouse mammary tumor virus (mmtv) promoter (Cre^mmtv^). The resulting mice are termed *Ptges* epithelial KO (*Ptges*^epi^). To induce mammary tumor development, control, *Ptges^–/–^*, and *Ptges*^epi^ mice were crossed with mice transgenic for the *Erbb2* (HER2/c-neu) oncogene carrying activating Val^664^ to Glu^664^ mutation also expressed under the control of mmtv promoter (The Jackson Laboratory). Genotype verification was performed by conventional PCR using primers listed in Supplemental Table 1. Her2/c-neu transgenic control, *Ptges^–/–^*, *Ptges^+/–^*, and *Ptges*^epi^ mice were sacrificed at 22 weeks for tumor onset and multiplicity study. Macroscopic tumors on all 10 mammary glands were counted at the necropsy, after which mammary glands 4 and 9 were harvested, fixed, paraffin embedded, sectioned, and H&E stained for microscopic evaluation of the number of tumors per gland.

All WT C57BL/6J were purchased from The Jackson Laboratory. Kras-LSL-G12D/+; Trp53-LSL-R172H/+, Pdx1-Cre (KPC), and Rosa-LSL-YFP (KPCY) mice (64, 65) were bred in-house, backcrossed for over 10 generations with C57BL/6J mice and assessed at the DartMouse Speed Congenic Core facility at the Geisel School of Medicine at Dartmouth College (Lebanon, New Hampshire, USA). *Ptger4^fl/fl^* and CAGGCre-ERTM mice on the C57BL/6J background were purchased from The Jackson Laboratory, crossed, and at 6–8 weeks of age treated with 100 mg tamoxifen (MilliporeSigma) for 5 days, to generate tamoxifen-inducible *Ptger4*-KO mice. Genotype verification of KPC, KPCY, and *Ptger4*-KO mice was performed by Transnetyx using real-time PCR with specific probes designed for each gene listed in Supplemental Table 1. For the survival studies, young, less than 10 weeks old, KPC and KPCY mice were monitored for the development of pancreatic tumors by palpation. After 10 weeks, all mice were monitored by ultrasound imaging (Vevo 2100 Imaging System with 55 MHz MicroScan transducer, Visual Sonics) once a week until tumor discovery, after which the tumor growth was monitored by biweekly ultrasound imaging. For tumor-bearing mice, endpoint criteria included tumor volume exceeding 500 mm^3^, severe cachexia or weakness, and inactivity.

### Tumor cell lines — generation, culture, and in vitro growth.

All mouse pancreatic tumor cell lines were generated from late-stage primary tumors from C57BL/6J background KPC and KPCY mice. Single-cell PDA clones were generated by limiting dilution from KPCY and KPC cell lines and tested by the Research Animal Diagnostic Laboratory (RADIL) at the University of Missouri (Columbia, Missouri, USA), using the Infectious Microbe PCR Amplification Test (IMPACT) II. Cells were cultured in high-glucose Dulbecco’s Modified Eagle’s medium (DMEM) with 10% FBS (Gibco), 2 mM glutamine, and 1 mg/mL gentamicin (complete DMEM). The clones were regularly tested using the MycoAlert Mycoplasma Detection Kit from Lonza. For in vitro growth analysis, the cells were plated either 5 × 10^4^ cells/well on 6-well plates with and without treatments and counted using a Celigo Image Cytometer (Nexcelom) or plated 1 × 10^5^ cells/flask in T25 flasks, trypsinized, and counted using Countess II cell counter (Life Technologies).

### RNA-seq, differential gene expression, GSEA, and patient survival analyses.

RNA samples were extracted from in vitro–cultured cell lines or YFP^+^ tumor cells sorted from s.c. implanted tumors or bulk tumors using the Qiagen RNeasy Micro Kit following the manufacturer’s instructions. RNA was sent out to a commercial company (Novogene) for library preparation and high-throughput sequencing using Illumina sequencers to generate paired-end 150-bp data. Fastq files were checked for quality using FastQC. Raw counts of gene transcripts were obtained using the alignment-independent tool, Salmon, using standard settings (66). The raw count matrix was subsequently imported into R-studio (R version 3.3.3) and used as input for DESeq2 following the vignette of the package for normalization and differential gene expression analysis (67). Salmon was also used to normalize and quantitate gene expression in transcripts-per-million (TPM) through quasi-alignment. Differentially expressed genes from the DESeq2 analysis were used as input for the GSEA MSigDB gene set enrichment analysis (68). Patient survival data were calculated based on the RNA-seq analysis of PDAC patients in The Cancer Genome Atlas (TCGA) dataset, using the Oncolnc tool (www.oncolnc.org; ref. 69).

### Q-PCR analysis for gene expression.

Total RNA from tumor cell lines was isolated (RNeasy, Qiagen) and reverse transcribed (Reverse Transcription Kit, Applied Biosystems) according to the manufacturers’ instructions. Real-time Q-PCR for *Ptges*, *Ptger4*, *18s*, and *Tbp* mRNA was performed using inventoried gene expression assays and TaqMan Universal PCR Master mix from Applied Biosystems (Supplemental Table 1). PCR products were detected in CFX384 (Bio-Rad), Vii A7, and QuantStudio 7 Pro (Applied Biosystems) real-time PCR systems. Results were analyzed using the comparative Ct method.

### Lentiviral transduction of tumor cells for CRISPR-mediated gene ablation.

sgRNAs against *Ptges* and *Ptger4* (Supplemental Table 1) were designed using the CRISPick tool (Broad Institute). The lentiCRISPR v2 (Addgene) containing sgRNAs or no sgRNA (empty vector), pVSVg, and psPAX2 lentiviral packaging plasmids (Addgene) were cotransfected into 293T cells (Clontech) using polyethylenimine (PEI, Polysciences). Lentiviral particles were collected from supernatants using a 0.45 μm PVDF filter, 48 hours after transfection. KPC cells were incubated for 72 hours with lentiviral particles in the presence of 8 μg/mL polybrene (MilliporeSigma) and then selected with 8 μg/mL puromycin (Invitrogen). Single cells were picked from the empty vector (control) and sgRNA (KO) transduced cell lines and expanded into clones. Deletion efficiencies were assessed by gene-specific Q-PCR analysis.

### Inducible gene KD in vitro and in vivo.

The sequences of shRNA targeting *Ptges* were obtained using splashRNA (http://splashrna.mskcc.org/) and the nucleotides were purchased from IDT and amplified using Platinum Pfx DNA Polymerase (Invitrogen). The PCR product was purified, cut with Xhol and EcoRI enzymes (NEB), gel purified, and cloned into Tet-ON RNAi vector LT3GEPIR expressing CFP (a gift from Scott Lowe’s lab at Memorial Sloan Kettering Cancer Center, New York, New York, USA). The control cells were transfected with an LT3GEPIR vector with no shRNA. After transfection, CFP^+^ single cells were sorted and expanded. *Ptges* expression was measured by Q-PCR in untreated and 1 μg/mL DOX–treated (Thermo Fisher Scientific) clonal cell lines in vitro. For in vivo tumor growth studies, control and *Ptges*-KD tumor cells were implanted s.c. into C57BL/6J mice. Starting on day 7 after implantation, tumor-bearing mice received 0.2 g DOX and 25 g D-sucrose (Thermo Fisher Scientific) in drinking water throughout the experiment.

### Ptger4 overexpression in E0771 mammary tumor cell line.

*The Ptger4* gene (sequence obtained from NCBI) was subcloned into the pTRPE vector (gift from Carl June’s laboratory at the University of Pennsylvania, Philadelphia, Pennsylvania, USA) coupled with T2A self-cleaving peptide sequence and a puromycin resistance gene (PuroR) for selection (pTRPE. EP4.T2A.PuroR). The empty vector, pTRPE_PuroR_Empty_269, was used to generate the control cell line. The integrity of the cloned plasmid was confirmed through restriction enzyme digestion and DNA sequencing to ensure accurate insertion and orientation of the gene of interest within the transfer vector. Lentiviral particles were produced via cotransfection of HEK293T cells with pTRPE.EP4.T2A.PuroR and pTRPE_PuroR_Empty_269 and packaging plasmids pMD2.G and psPAX2 using Lipofectamine 3000 (Thermo Fisher Scientific). Lentiviral particles were collected 48 hours after transfection using a 0.45 μm pore-size filter and concentrated via ultracentrifugation. E0771 cells were incubated for 48 hours with lentiviral particles in the presence of 8 μg/mL polybrene and then selected with 4 μg/mL puromycin and maintained in 2 μg/mL puromycin. Single cells were picked from the pTRPE.EP4.T2A.PuroR and pTRPE_PuroR_Empty_269 transduced cell lines and expanded into clones. Overexpression efficiencies were assessed by gene-specific Q-PCR analysis.

### hPTGES KI in mouse Ptges-KO cell line.

The h*PTGES* CDS was cloned from a human *PTGES* ORF vector (Applied Biological Materials) and inserted into a pCDH-EF1-FHC vector (Addgene). PAM sequences in h*PTGES* were point mutated using a Q5 site-directed mutagenesis kit (NEB) to prevent gene ablation when inserted into the m*Ptges*-KO cell line expressing Cas9 and sgRNA against m*Ptges*. The 6419c5 *Ptges*-KO D6 (m*Ptges*-KO) cell line was transduced with either the h*PTGES* gene–carrying vector to generate the m*Ptges*-KO cell line with h*PTGES* KI (m*Ptges* KO/h*PTGES* KI), or the empty pCDH-EF1-FHC vector to generate control cell line (m*Ptges* KO/KI EV). m*Ptges* absence and h*PTGES* overexpression were confirmed by Q-PCR. Restored PGE_2_ production by m*Ptges* KO/h*PTGES* KI was confirmed using PGE_2_ ELISA (Cayman Chemical).

### PGE_2_ measurements.

Control and KO cell lines were plated at 50,000 cells/well on 6-well plates and cultured as described in *Tumor cell lines* — *generation, culture, and in vitro growth*. On day 2, the regular culture medium was replaced with no-FBS medium and cells were allowed to synchronize overnight, after which the cells received complete medium with 10% FBS. After 24-hour incubation, media were harvested and stored at –80°C. Spot urine was collected from untreated, mPGES-1 inhibitor–, and 0.2 g DOX–treated C57BL/6J mice and stored at –80°C. PGE_2_ levels in media and urine samples were measured by ELISA according to the manufacturer’s (Cayman Chemical) instructions.

### Implantation of tumor cells and tumor measurements.

Control, *Ptges*-KO, and *Ptger4*-KO cultured tumor cells were dissociated into single cells with 0.25% trypsin (Gibco), washed with serum-free DMEM twice, and counted in preparation for s.c. or orthotopic implantations. For tumor growth and survival studies, 1 × 10^5^ to 3.0 × 10^5^ tumor cells in 100 mL sterile PBS were implanted s.c. into the flanks of 6- to 8-week-old host mice. For tumor growth kinetics, tumors were measured twice a week. Tumor length and width were measured with calipers, and tumor volumes were then calculated as length × width^2^/2. Tumor volumes of 1 cm^3^ were used as an endpoint for survival analysis. For fixed time point tumor analysis, 5 × 10^5^ or 1 × 10^6^ cells/100 mL sterile PBS were injected. Tumors were harvested 7–11 days after implantation. Tumor growth and host survival experiments were performed at least twice.

For orthotopic injections, mice were anesthetized, and an incision was made over the upper left quadrant of the sterilized abdomen. Control and *Ptges*-KO cells (both 1 × 10^5^ cells/50 mL DMEM) were injected into the tail of the pancreas. A cotton swab was used to absorb bleeding and ensure that cells did not leak into the peritoneal cavity. The successful injection was confirmed by liquid bleb formation at the site of injection. The peritoneum and incision were closed with sterile 4-0 violet braided polyglycolic acid sutures (CP Medical Inc). For ultrasound-guided orthotopic injections, 2 × 10^5^ control and *Ptger4*-KO cells per 25 mL DMEM were injected as described previously (70). Orthotopic tumor growth was assessed by weekly ultrasound imaging and measurements. Orthotopic tumor–bearing mice were either sacrificed at a fixed time point or followed for growth kinetics and sacrificed when tumors were 500 mm^3^ or larger.

### Cell depletions and treatments.

The depleting and monoclonal antibodies (mAb) in 100 μL sterile PBS and chemotherapy drugs were administered via intraperitoneal (i.p.) injections. CD4^+^ and CD8^+^ T cells were depleted using 200 mg endotoxin-free anti-CD4 (clone GK1.5, BioXcell) and anti-CD8 (clone 2.43, BioXcell) antibodies, 3 days before tumor implantation and twice a week thereafter, for the duration of the experiment. Control groups received rat IgG2b isotype control antibody (clone LTF-2, BioXcell). T cell depletion in the peripheral blood was confirmed by flow cytometry.

An αPD-1 mAb (200 μg; clone RMP 1-14, BioXcell) was administered on days 1, 4, 7, 10, 13, and 16 after enrollment. A single dose of 100 μg agonistic CD40 rat anti–mouse IgG2a mAb (aCD40; clone FGK45, endotoxin-free, BioXcell) was administered on day 3. EP4 antagonist (αEP4, 10 mg/kg) ONO-AE3-208 (*K*_i_ = 1.3 nM, Cayman Chemical) was administered i.p. in 100 μL sterile PBS daily for 14 days to s.c. tumor–bearing mice and 21 days to KPC tumor–bearing mice. For dual αEP2/αEP4 treatment, at the enrollment, tumor-bearing mice were placed on a medicated diet (Envigo) containing ONO-AE3-208 and EP2 antagonist PF-04418948 (IC_50_ = 16 nM; Cayman Chemical) at 10 mg/kg for both, for the duration of the experiment. Nab-paclitaxel (Abraxane, Celgene) and gemcitabine (Novaplus) were purchased from the Hospital of the University of Pennsylvania and administered i.p. at 120 mg/kg. After the first treatment with both drugs, mice continued receiving only gemcitabine weekly until the end of the experiment. All treatments were started when tumor volumes were not greater than 50 mm^3^.

### Flow cytometry of implanted tumors and tumor-draining lymph nodes.

Tumors were digested in 1 mg/mL collagenase with protease inhibitor (MilliporeSigma) and filtered through a 70 μm cell strainer. Tumor-draining lymph nodes were mechanically dissociated and filtered. Cells from each tumor or lymph node were counted, resuspended up to 1 × 10^6^ cells/100 μL FACS buffer, and 100 μL of from each sample transferred to a 96-well plate for staining. Samples were stained for cell-surface molecules and then fixed and permeabilized (eBioscience Intracellular Fixation & Permeabilization Buffer Set, Invitrogen) and further stained for intracellular targets. Flow cytometry was performed using an LSRFortessa or FACSymphony A3 and data were analyzed using FlowJo v10.8 software (BD Biosciences). Flow cytometric analysis of s.c. implanted tumors and tumor-draining lymph nodes was performed at least twice. Molecular targets, respective antibodies, and cell type markers are listed in Supplemental Table 2.

### Immunohistochemistry.

For CD3 and Ly-6G stainings, tumor tissues were fixed in Zn-formalin for 24 hours and embedded in paraffin. Deparaffinized tissue sections were stained with goat anti–mouse CD3 (Santa Cruz Biotechnology) and rat anti–mouse Ly-6G (STEMCELL Technologies) antibodies. Slides were scanned with an Aperio CS-Oscanner and whole slide images were visualized and analyzed using Aperio ImageScope software (Leica Biosystems).

### Released and intracellular cAMP measurements.

For released cAMP measurements, 4662 MD7 cells were plated 5 × 10^4^ cells/well on 6-well plates in 2 mL complete DMEM. After 24 hours, the medium was replaced with fresh medium with or without αEP4 ONO-AE3-208. Media were harvested 24 hours later and stored at –80°C. For intracellular cAMP measurements, 6419c5 EV8 and 6419c5 *Ptger4*-KO E10 cells were pretreated in vitro for 10 minutes with 100 μM phosphodiesterase inhibitor Ro 20-1724 (Tocris Bioscience) followed by an additional 10-minute incubation with Ro 20-1724 plus 1 μM PGE_2_, after which the cells were lysed with 0.1 M HCl and stored at –80°C until being analyzed with a cAMP ELISA kit (Cayman Chemical) according to the manufacturer’s instructions.

### Cell viability assay.

The assay was performed as previously described (71). Tumor cells (3 × 10^3^ to 5 × 10^3^) were seeded in a 96-well plate. The next day, cells were treated with 1 μg/mL of cycloheximide (Cell Signaling Technology) with 0.2 μg/mL IFN-γ (Peprotech) and indicated concentrations of TNF-α (BioLegend). Two days later, the media were replaced with fresh DMEM, and cell viability was measured by CellTiter-Glo (Promega). Data were normalized to the control group without TNF-α treatment. Nonlinear regression curves were determined using GraphPad Prism 9.

### Ex vivo cytokine and adenosine measurements.

Female C57BL/6J mice were injected with 1 × 10^6^ tumor cells subcutaneously on the flank. On day 11 after implantation, the tumors were harvested, weighed, washed with PBS, minced, and placed in the inserts of a 24-well Corning Transwell plate and cultured in complete DMEM at 37°C and 5% CO_2_. After 24 hours, the media were replaced with fresh complete DMEM containing 1 μM PGE_2_ (Cayman Chemical) and 10 μM adenosine deaminase inhibitor EHNA (MilliporeSigma), and the tumors were cultured in a hypoxia chamber at <1% O_2_. After 24 hours, the media were harvested, aliquoted, and stored at –80°C. For cytokine and chemokine analysis using Proteome Profiler Mouse XL Cytokine Array kit (Biotechne/R&D Systems), aliquots of samples in the same group (7 control and 8 *Ptger4*-KO E10 samples) were pooled. The Cytokine Array membranes were imaged using ImageQuant LAS 4000 (GE Healthcare Life Sciences) and quantified with ImageQuant TL software (Cytiva).

Analysis using Legendplex Mouse Inflammation and Proinflammatory Chemokine Panel assays (BioLegend), was performed on individual, non-pooled samples, according to manufacturer instructions. For adenosine measurements, 100 μL aliquots of media were spiked with isotopically labeled adenosine and extracted with 400 μL of ice-cold methanol on ice. After centrifuging at 18.0*g* for 5 minutes at 4°C, the methanol supernatant was dried under nitrogen at 4 °C in a 96-well plate, reconstituted in 500 μL of deionized water, and analyzed on an Agilent 1290 Infinity UHPLC/6495B triple quadruple mass spectrometer. Calibration curves with standard adenosine were similarly prepared and data were processed using Agilent Mass Hunter quantitation software.

### p-Smad3 and β-actin Western blot analysis.

Control and *Ptger4*-KO E10 cells were plated 5 × 10^5^ cells/flask in T25 flasks, in complete DMEM. After overnight incubation at 37°C, the cells were switched to fresh complete DMEM with 1 μM PGE_2_. After 30 minutes, 5 ng/mL TGF-β was added to the media. After 15 minutes, the media were removed, and cells were washed and lysed with RIPA buffer with protease inhibitor (MilliporeSigma). An aliquot of each sample was used to quantify the protein concentration using the Pierce BCA Protein Assay Kit (Thermo Fisher Scientific). Equal protein amounts of reduced samples were loaded on 4%–12% NuPAGE Bis-Tris gels for electrophoresis. After a 3-hour run at 90 V, the samples were transferred to a nitrocellulose membrane (Invitrogen), blocked with 5% milk for 1 hour, and incubated with primary antibodies overnight at 4°C, followed by 30-minute incubation with horseradish peroxidase–conjugated secondary antibody and 1-minute incubation with ECL reagent (Supplemental Table 3). The membranes were imaged using ImageQuant LAS 4000 (GE Healthcare Life Sciences) and quantified with ImageQuant TL software (Cytiva).

### Software.

Prism software and R were used for data processing, statistical analysis, and result visualization (http://www.graphpad.com). The R language and environment for statistical computing and graphics (https://www.r-project.org) was utilized in this study for the bioinformatics and statistical analyses of RNA-seq data and survival comparison between multiple groups. The R packages were obtained from Bioconductor (https://www.bioconductor.org) and CRAN (https://cran.r-project.org/web/packages/available_packages_by_name.html).

### Statistics.

Statistical comparisons between 2 groups were performed using Student’s unpaired *t* test. For comparisons between multiple groups, 1-way ANOVA with Tukey’s HSD post hoc test and 2-way ANOVA with main or mixed-effect analysis were used. For survival comparison between 2 groups, log-rank Kaplan-Meier and Gehan-Breslow-Wilcoxen survival tests were performed in GraphPad Prism 7 and Oncolnc (http://www.oncolnc.org/). Survival curve comparisons between multiple groups were performed using the survival package in R. For all figures, a *P* value of less than 0.05 was considered statistically significant: *P < 0.05, **P < 0.01, ***P < 0.001, and ****P < 0.0001. NS denotes not significant.

### Study approval.

All animal procedures were conducted in accordance with the NIH *Guide for the Care and Use of Laboratory Animals* (National Academies Press, 2011) and were approved by the Institutional Animal Care and Use Committee of the University of Pennsylvania.

### Data resources.

The RNA-seq data were deposited in the NCBI’s Gene Expression Omnibus database – GEO submission GSE275728 (NCBI tracking system 24791746).

### Data, reagent, and resource availability.

Underlying data for all figures are available in the Supporting Data Values file. Requests for additional data, supporting analytic code, and reagents should be directed to the corresponding authors Robert H. Vonderheide and Ben Z. Stanger.

## Author contributions

NM, RHV, BZS, GAF, and EMS conceived the project. NM, IKK, BZS, and RHV developed the methods. NM and IKK analyzed the data. JWT and NJ developed the software. NM, IKK, CA, LQW, NC, EYS, and JEH performed experiments. NM, IKK, RHV, BZS, JWT, EC, KL, VRD, and LCC curated the data. NM, IKK, CA, BZS, and RHV wrote the manuscript. All authors approved the manuscript.

## Supplementary Material

Supplemental data

Unedited blot and gel images

Supporting data values

## Version 1 09/19/2024

In-Press Preview

## Version 2 11/08/2024

Electronic publication
