# Supplementary material for: Pivotal roles for cancer cell–intrinsic mPGES-1 and autocrine EP4 signaling in suppressing antitumor immunity
Source: JCI Insight. 2024 Nov 8;9(21):e178644. doi: 10.1172/jci.insight.178644 (PMC11601572; doi:10.1172/jci.insight.178644)
Supplement: Supplemental data [file jciinsight-9-178644-s161.pdf]

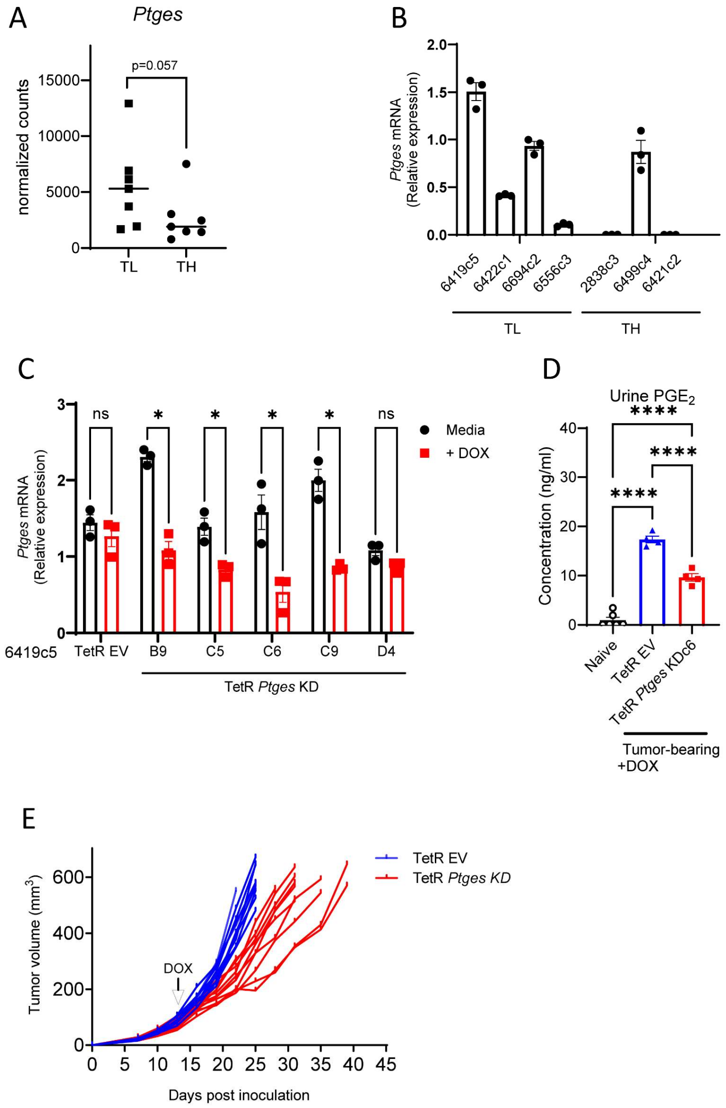

Supplemental Figure 1. A. *Ptges* transcript counts in bulk RNA-seq of T cell low (TL) and T cell high (TH) KPC or KPCY tumors (n=7). **B.** *Ptges* mRNA expression in TL and TH clones by Q-PCR. **C.** *Ptges* mRNA expression in TetR EV and TetR *Ptges* KD single-cell clones with or without doxycycline (DOX) treatment *in vitro* (n=3). D. PGE_2 measurements in the urine of non-tumor-bearing (naïve) and doxycycline treated (DOX) TetR EV and TetR *Ptges* KDc6 tumor-bearing mice. E. Individual growth curves of TetR EV and TetR *Ptges* KD tumors with DOX administration initiated 14 days post-implantation. Data: (A) median and (B, C, and D) mean +/- SEM. Statistical analysis: (A) One-tailed unpaired t-test, (C) Multiple T-tests, and (D) One-way ANOVA with Tukey's test for multiple comparisons. $p<0.05$ was considered statistically significant, and * $p<0.05$, **** $p< 0.0001$.

A

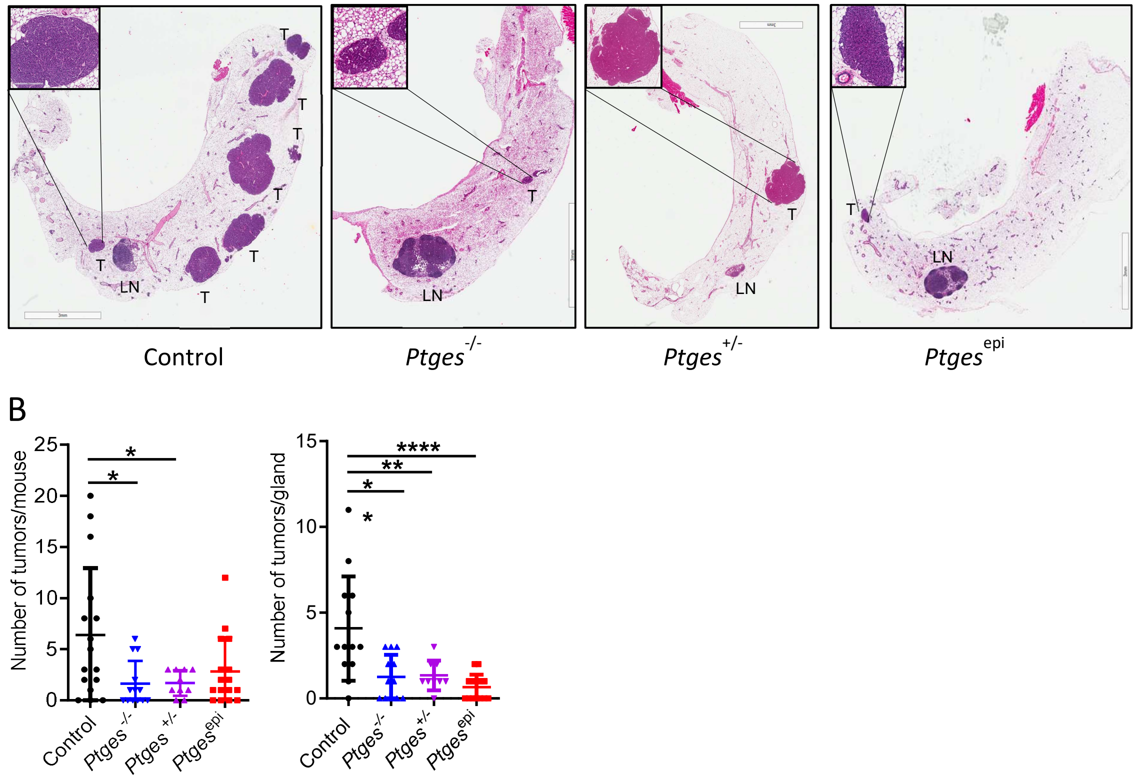

Supplemental Figure 2. **A**. Whole slide images of H&E stained #9 abdominal mammary glands harvested from 22-week-old *Her2/neu* transgenic mice with no deletion of floxed (fl) *Ptges* gene (Ptges $^{fl/fl}$) or lacking both or one copies of *Ptges* globally (*Ptges*$^{-/-}$ and Ptges$^{+/-}$, respectively) or both copies in mammary epithelial cells only (*Ptges*^{epi}). **B**. Quantification of tumors per mouse (left) or per #9 gland (right, n=10-16). Data: (B) mean +/- SD. Statistical analysis: (B) Ordinary one-way ANOVA with Tukey's multiple comparison test. For all panels, p<0.05 was considered statistically significant, and * p<0.05, ** p<0.01, and **** p< 0.0001.

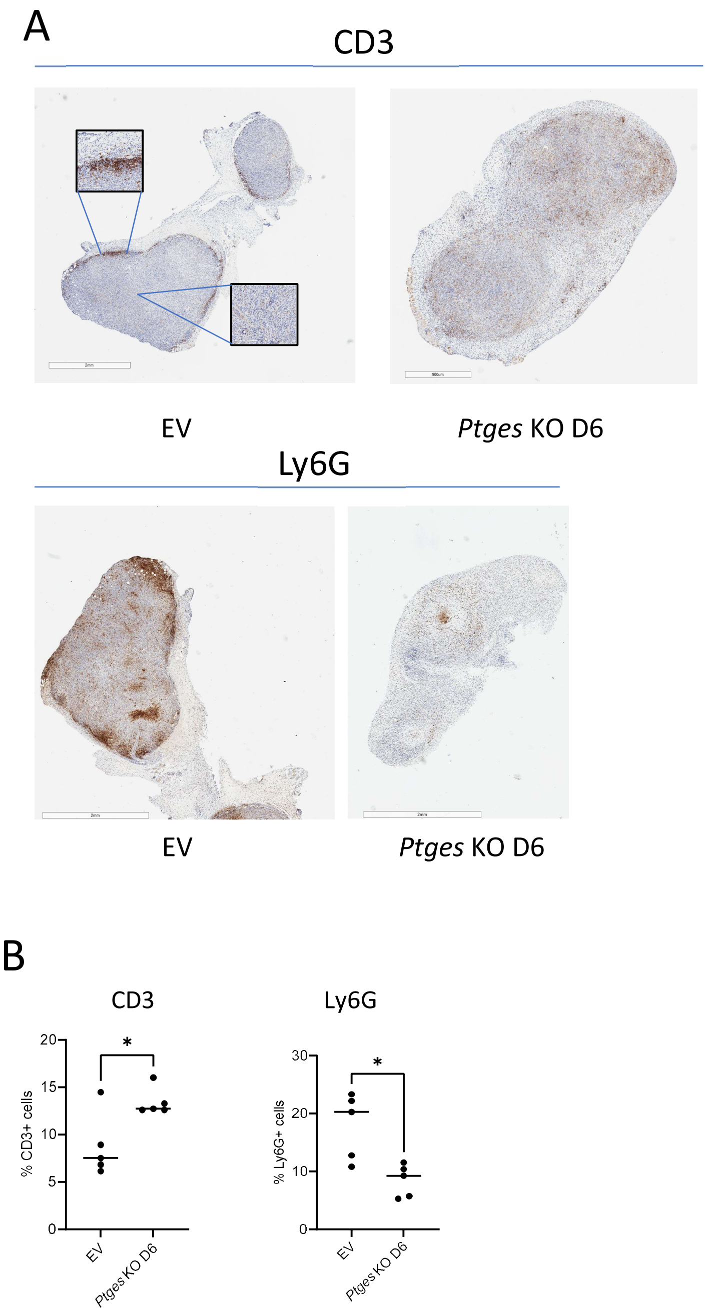

Supplemental Figure 3. A. Immunohistochemistry for CD3 and Ly6G performed on control and *Ptges* KO tumors harvested 7 days post sc implantation. Brown staining represents cells stained for indicated markers. **B**. Quantification of stainings in (A). Quantification was done across the whole tumor surface and shown as percent of total cells positive for the marker (n=5). Data: (B) median. Statistical analysis: (B) Two-tailed unpaired t-test. For all panels, $p<0.05$ was considered statistically significant, and * $p<0.05$.

A

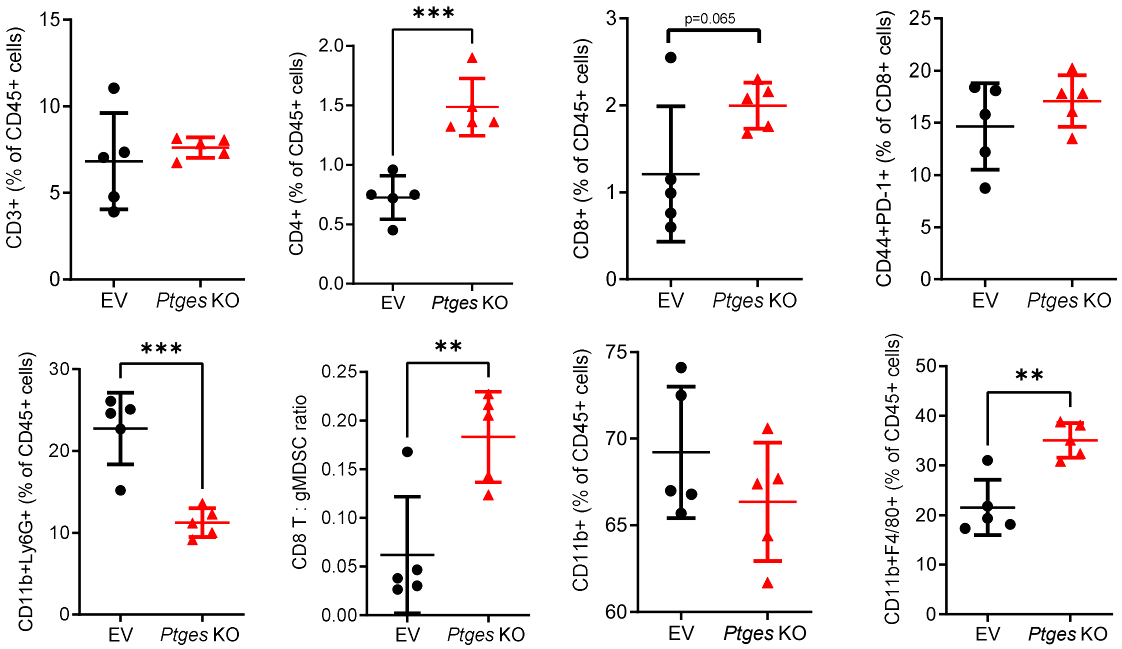

B

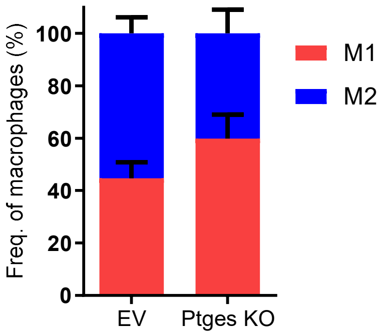

Supplemental Figure 4. A. Flow cytometry analysis of sc implanted EV and *Ptges* KO 6694c2 PDAC tumors on day 7 post-implantation (n=5). **B**. Proportions of M1 (F4/80+ CD206-MHC II^{high}) and M2 (F4/80+CD206+MHC II^{inter}) macrophages as a percentage of total macrophages in sc implanted EV and *Ptges* KO 6694c2 tumors (flow cytometry, day 7 post-implantation, n=5). Data: (A) median and (B) mean +/- SD. Statistical analysis: Two-tailed unpaired t-test. For all figures, p<0.05 was considered statistically significant, and ** p<0.01, and *** p< 0.001.

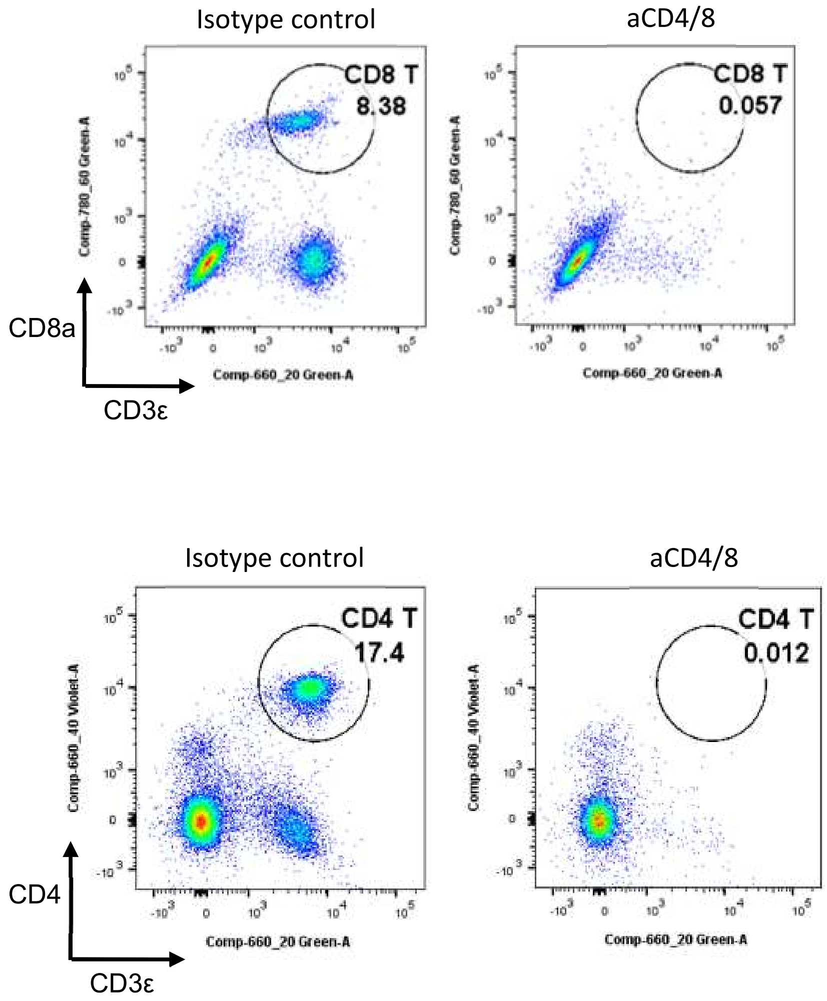

Supplemental Figure 5. Flow cytometry analysis for T cells in blood collected from hosts receiving either isotype control or anti-CD4 and anti-CD8 depleting antibodies.

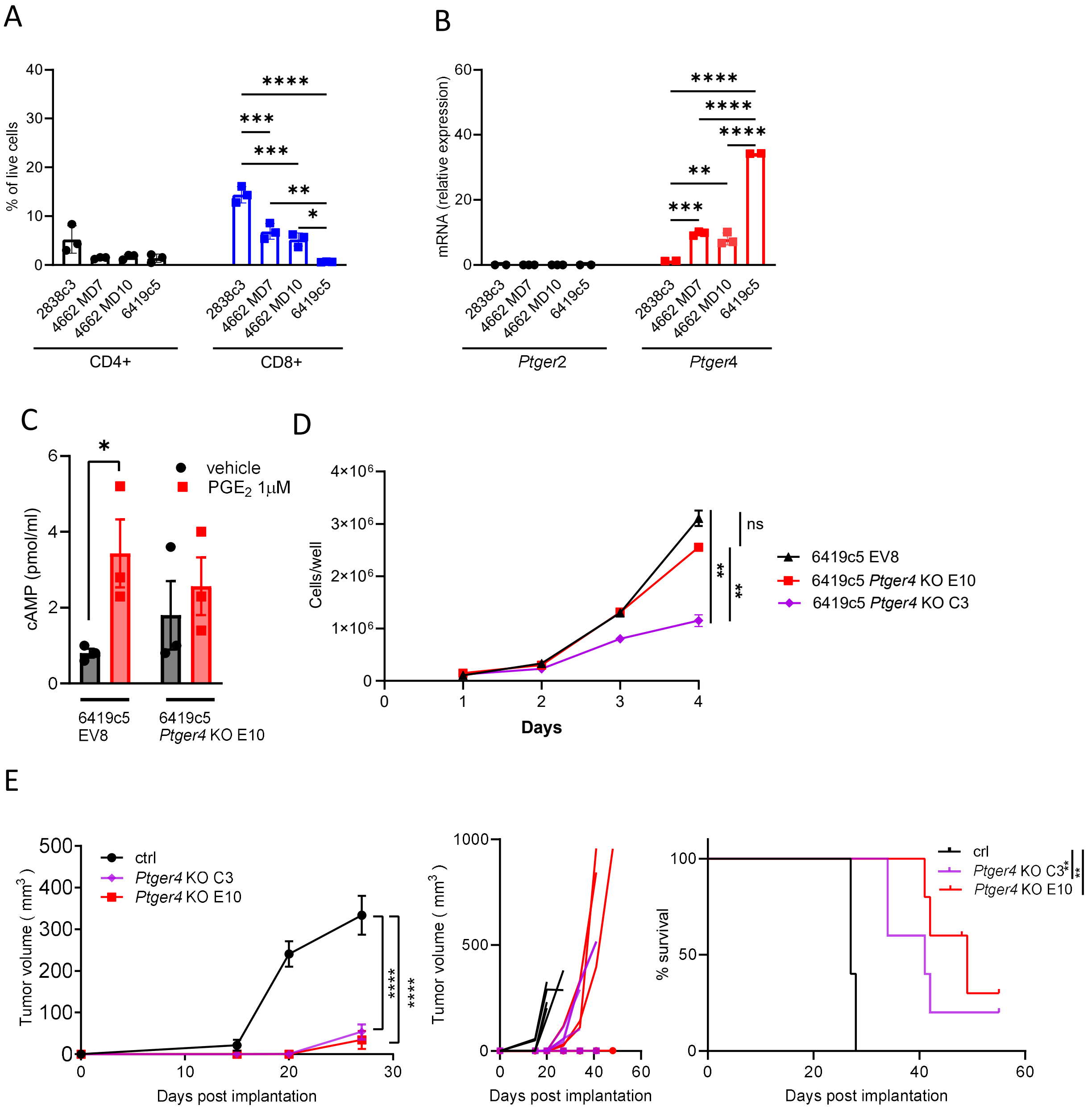

Supplemental Figure 6. A. Proportions of CD8+ and CD4+ T cells in tumors from indicated sc implanted clonal PDAC cell lines by flow cytometry. **B**. Relative expression of *Ptger2* and *Ptger4* receptors in indicated clonal PDAC cell lines by Q-PCR. **C**. Intracellular cAMP measurements in cultured 6419c5 EV8 and 6419c5 *Ptger*4 KO E10 clones treated with 1μM PGE_2 (n=3). **D**. Growth of control EV and *Ptger4* KO clonal cell lines *in vitro*. **E.** Growth of orthotopically implanted control and *Ptger4 KO* clonal cell lines (n=5, left- cumulative growth, middle – individual tumor growth curves) and host survival (right). Data: (A) mean +/- SD, (B, C, D, and E, left) mean +/- SEM. Statistical analysis: (A and B) One-way ANOVA with Tukey's multiple comparison test, (C) Multiple unpaired t-tests, (D and E, left) Two-way ANOVA with Tukey's multiple comparison test for main-effects analysis, (E, right) survival package in R. For all figures, $p<0.05$ was considered statistically significant and * $p<0.05$, ** $p<0.01$, *** $p< 0.001$, and **** $p< 0.0001$ and non-significant (ns).

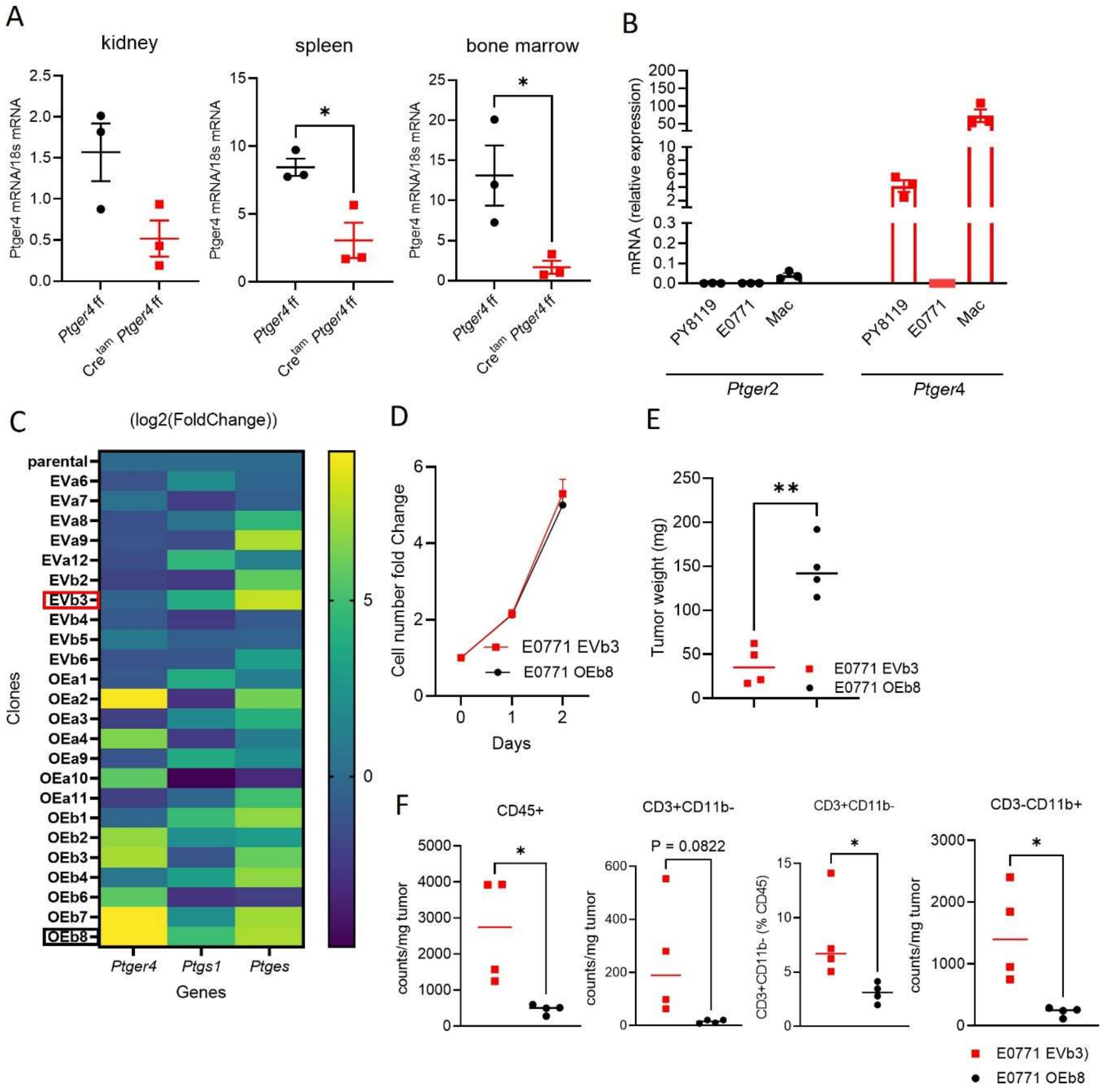

Supplemental Figure 7. A. *Ptger4* expression by Q-PCR in indicated tissues harvested from control (*Ptger4* flox/flox (ff)) and *Ptger4* KO (Cre^{tam}*Ptger4* ff) mice. **B.** *Ptger2* and *Ptger4* mRNA expression by Q-PCR in indicated mammary tumor cell lines and macrophages (mac) used as a positive control for *Ptger4* expression (n=3). **C.** Heatmap of *Ptger4* mRNA expression in single cell clones generated from E0771 EV and E0771 OE cell lines (the vertical bar indicates log2(fold change)). **D.** *In vitro* growth of E0771 EVb3 and E0771OEb8 cell lines (n=6). **E.** Tumor weights 9 days post orthotopic implantation of E0771 EVb3 and E0771 OEb8 mammary tumor cell lines (n=4, one of two experiments with similar results shown). **F.** Flow cytometry analysis of E0771 EVb3 and E0771 OEb8 tumors 9 days post-implantation (n=4). Data: (A, B, and D) mean +/- SEM, (E and F) median. Statistical analysis: (A, E, and F) Two-tailed unpaired t-test. For all panels, p<0.05 was considered statistically significant, and * p<0.05, **p<0.01.

A

	padj	NES
HALLMARK_TNFA_SIGNALING_VIA_NFKB	0.004973145	2.50366
HALLMARK_INTERFERON_GAMMA_RESPONSE	0.004973145	2.36287
HALLMARK_INTERFERON_ALPHA_RESPONSE	0.004973145	2.08542
HALLMARK_ALLOGRAFT_REJECTION	0.004973145	1.94943

Bulk tumor

Ptger4 KO vs EV

	padj	NES
HALLMARK_TNFA_SIGNALING_VIA_NFKB	0.009442871	2.297181
HALLMARK_INTERFERON_ALPHA_RESPONSE	0.009442871	2.140404
HALLMARK_INTERFERON_GAMMA_RESPONSE	0.009442871	2.106577
HALLMARK_UV_RESPONSE_DN	0.009442871	1.820504

YFP+ *in vivo*

Ptger4 KO vs EV

	padj	NES
HALLMARK_TNFA_SIGNALING_VIA_NFKB	0.008403361	2.268812
HALLMARK_EPITHELIAL_MESENCHYMAL_TRANSITION	0.008403361	2.009039
HALLMARK_MYOGENESIS	0.008403361	1.927442
HALLMARK_COAGULATION	0.008403361	1.901939

Bulk tumor

Ptges KO vs EV

	padj	NES
HALLMARK_TNFA_SIGNALING_VIA_NFKB	0.008668516	1.855098
HALLMARK_UV_RESPONSE_DN	0.008668516	1.626388
HALLMARK_COAGULATION	0.017088175	1.455845
HALLMARK_PROTEIN_SECRETION	0.050944165	1.340295

YFP+ *in vivo*

Ptges KO vs EV

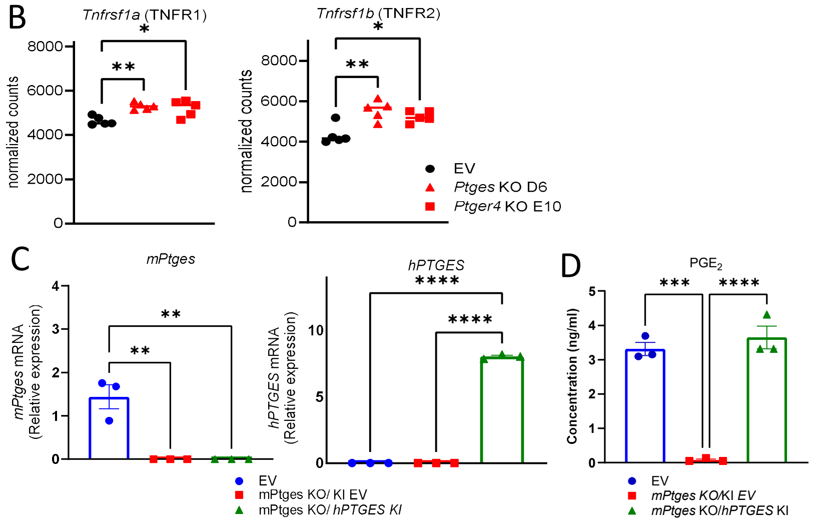

Supplemental Figure 8. A. Top upregulated pathways by RNA-seq GSEA analysis in indicated comparisons (n=4-5). **B.** Transcript abundance of TNFα receptor genes by RNA-seq in bulk control EV, *Ptger4* KO, and *Ptges* KO tumors, 11 days post-implantation (n=5). **C.** Mouse *Ptges* (m*Ptges*) and human *PTGES* (h*PTGES*) gene expression by Q-PCR in the indicated cell lines (n=3). **D.** PGE_2 concentration measured in the culture media of indicated cell lines by ELISA (n=3). Data: (B) median, (C and D) mean +/- SEM. Statistics: (B, C, and D) Ordinary one-way ANOVA with Tukey's multiple comparison test. $p<0.05$ was considered statistically significant and * $p<0.05$, ** $p<0.01$, *** $p< 0.001$, and **** $p< 0.0001$.

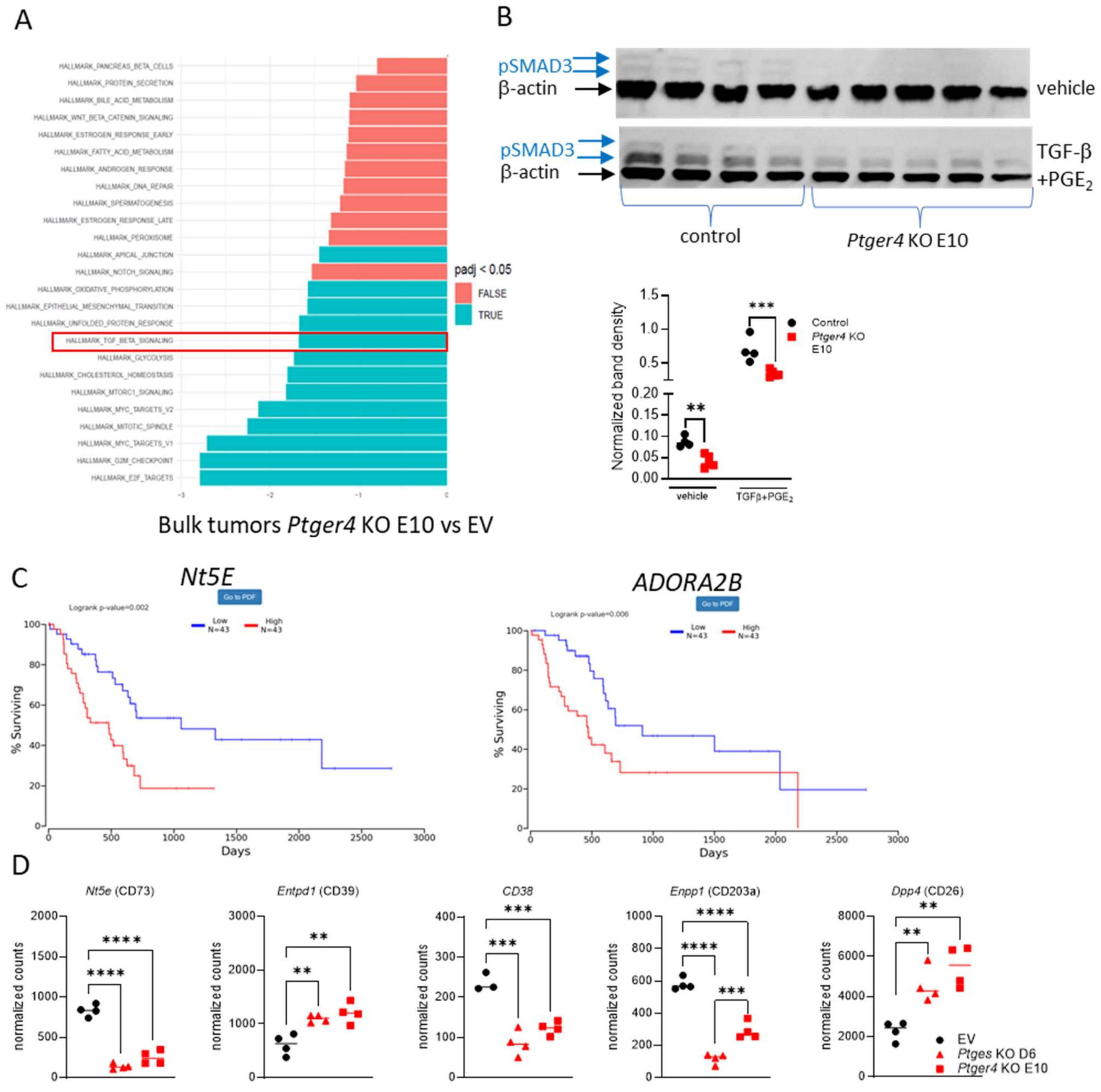

Supplemental Figure 9. A. Top downregulated pathways by RNA-seq GSEA analysis in bulk sc *Ptger4* KO compared to the control EV tumors, 10 days post-implantation (n=5). **B.** Western blot analysis for phosphorylated SMAD3 (blue arrows indicate bands corresponding to phospho(p) S423 and S425 peptides) in EV and *Ptger4* KO tumor cells, treated *in vitro* either with vehicle or TGF-β and PGE_2 (n=4, top panel - gel images, bottom panel - band density quantification relative to β-actin). **C**. Survival of pancreatic cancer patients from the TCGA dataset stratified into upper and lower quartiles of the expression of the indicated gene (n=43). **D**. Transcript abundance of indicated genes by RNA-seq in YFP+ cells flow-sorted from control EV, *Ptger4* KO, and *Ptges* KO tumors, 11 days post-implantation (n=4). Data: (B and D) median. Statistics: (B) Two-tailed unpaired t-test, (C) Log-rank analysis performed in Oncolnc.org, and (D) Ordinary one-way ANOVA with Tukey's multiple comparison test. For all figures, p<0.05 was considered statistically significant, and * p<0.05, ** p<0.01, *** p< 0.001, and **** p< 0.0001.

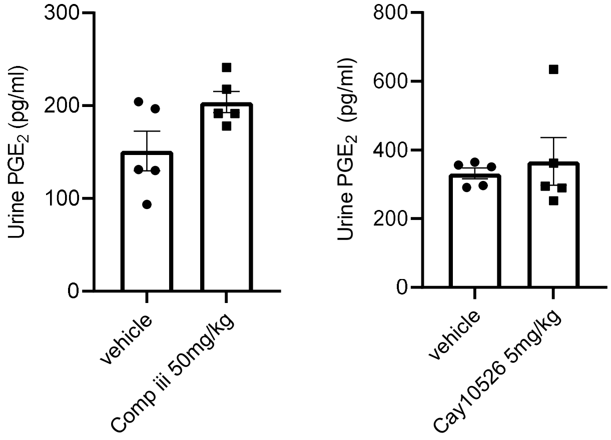

Supplemental Figure 10. PGE2 measurements by ELISA in spot urine collected from wild-type C57Bl/6 mice treated with either vehicle or indicated mPGES-1 inhibitor for 5 days (n=5). Data: mean +/- SD. Statistics: Two-tailed unpaired t-test.

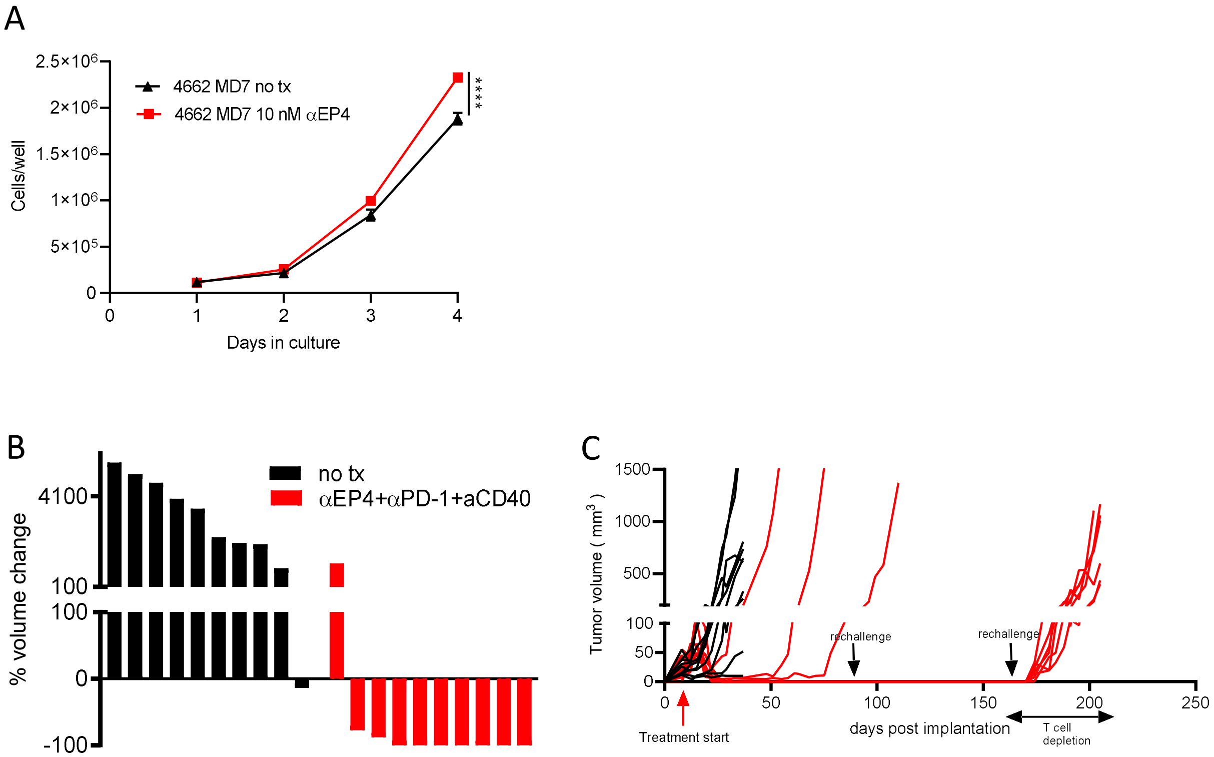

Supplemental Figure 11. A. Growth of 4662 MD7 clone *in vitro* with and without 10 nM EP4 antagonist ONO-AE3-208 (n=6). **B.** Volume change of sc implanted 4662 MD10 clonal PDA cell line with and without indicated treatment on day 37 post-implantation (28th day of treatment, left, n=10). Right – individual growth curves of rechallenge tumors implanted in cured hosts from the left panel. The red vertical arrow indicates the start of the treatment, the black vertical arrows indicate rechallenge days, and the black horizontal arrow indicates the period of T cell depletion. Data: (A) mean +/- SD. Statistics: (A) Two-way ANOVA with main-effects analysis; p<0.05 was considered statistically significant and **** p< 0.0001.

PCR Primers and sgRNAs		
Target	Company	Catalog Number
18s ribosomal RNA (18s)	Life Technologies	Mm04277571_s1Rn18s;R
Tbp forward	agaaacaatccagactagcagca	
Tbp reverse	gggaacttcacatcacagctc	
Ptger4, exon 1-2	Life Technologies	Mm01303635_g1
Ptger4, exon 1-2	Life Technologies	Mm00436052_g1
Ptger4, exon 2-3	Life Technologies	Mm00436053_m1
Ptger2	Life Technologies	Mm_00436051_m1
COX-1 (Ptgs1)	Life Technologies	Mm00477214_m
COX-2 (Ptgs2)	Life Technologies	Mm00478374_m1
Ptges	Life Technologies	Mm00452105_m1Ptges
Target	Sequence	
Ptges KO^{gl} (wild type allele) forward	tcccaggtgttgggatttagacg	
Ptges KO^{gl} (wild type allele) reverse	tagctggctgtactgtttgttgc	
Ptges KO^{epi} forward	actccagtactgagccagctgc	
Ptges KO^{epi} reverse	tgctacttccatttgtcacgtcc	
Cre^{mmtv} forward	tcgatgcaacgagtgatgagg	
Cre^{mmtv} reverse	acgaacctggtcgaaatcagt	
HER2/neu forward	ggacatccaggaagttcagggttac	
HER2/neu reverse	acaggagccagttggttattctgg	
Kras-LSL-G12D forward	gtcgagggacctaataacttcgt	
Kras-LSL-G12D reverse	acgctataccctgtggacaca	
Kras-LSL-G12D reporter	aagttatgtcgacaagctc	
p53-LSL-R172H forward	gtcgagggacctaataacttcgt	
p53-LSL-R172H reverse	agaggctggatgtgtaagaaaatgt	
P53-LSL-R172H reporter	atgtcgagtctattgcctttc	
Pdx-Cre forward	ttaatccatattggcagaacgaaaacg	
Pdx-Cre reverse	caggctaagtgccttctctaca	
Pdx-Cre reporter	cctgcggtgctaacc	
Rosa-LSL-YFP forward	caccctcgtgaccacctt	
Rosa-LSL-YFP reverse	ggtagcgggcgaagca	
Rosa-LSL-YFP reporter	ctgcaggccgtagccg	
CAGGCre-ERTM forward	ttaatccatattggcagaacgaaaacg	
CAGGCre-ERTM reverse	caggctaagtgccttctctaca	
CAGGCre-ERTM reporter	cctgcggtgctaacc	
Ptger4 flox forward	caaacccagggcgataagct	
Ptger4 flox reverse	gggttattgaatatgatcggaattcatcga	
Ptger4 flox reporter	aagcttgatatcgaattcaac	
Ptger4 WT forward	gtcagcagagcggtgct	
Ptger4 WT reverse	tcggtagctactcattaactgtgact	
Ptger4 WT reporter	aacggatccagaccttg	
Ptges sgRNA-1	ggaatgagtacacgaagccg	
Ptges sgRNA-2	gaggatgcgctgaaacgtgg	
Ptger4 sgRNA-1	cgacttgcacaatactacga	
Ptger4 sgRNA-2	ccagttatatcagccaaacg	

Supplemental Table 1

Flow Cytometry Antibodies			
Target	Company	Catalog Number	Dilution
CC3	Cell Signaling	9978S	1:50
CD3	BD Optibuild	740268	1:100
CD3	BioLegend	100241	1:100
CD3	BioLegend	100310	1:100
CD4	BD Biosciences	563790	1:100
CD4	BD Horizon	612900	1:100
CD4	BioLegend	128030	1:100
CD8	BD Biosciences	564920	1:100
CD8	BioLegend	100706	1:50
CD8	BioLegend	100740	1:100
CD11a	BD Optibuild	740340	1:100
CD11b	BD Biosciences	550993	1:200
CD11b	BioLegend	101208	1:100
CD11b	BioLegend	101251	1:100
CD11c	BioLegend	117330	1:20
CD11c	BioLegend	117336	1:100
CD11c	BioLegend	117334	1:100
CD26	BioLegend	137810	1:100
CD38	BD Biosciences	741514	1:100
CD39	ThermoFisher	12-0391-82	1:100
CD39	BioLegend	143806	1:100
CD39	BioLegend	143810	1:20
CD39	BioLegend	143812	1:100
CD40	BD Biosciences	562847	1:100
CD44	BioLegend	103036	1:100
CD44	BioLegend	103059	1:100
CD44	BD Biosciences	563058	1:100
CD45	BioLegend	103112	1:100
CD45	BioLegend	103128	1:100
CD47	BD Optibuild	740744	1:100
CD62L	BioLegend	104428	1:100
CD64	BioLegend	139323	1:100
CD73	BioLegend	127215	1:100
CD80	BioLegend	104722	1:20
CD86	BioLegend	105016	1:100
CD103	BioLegend	121430	1:100
CD120a	BioLegend	113006	1:50
CD206	BioLegend	141719	1:100
CD279	BioLegend	135214	1:100
CD335	BioLegend	137604	1:100
CTLA4	BioLegend	106306	1:20
CTLA4	BD Biosciences	564332	1:100
ENPP1	BioLegend	149203	1:100
Eomes	ThermoFisher	25-4875-82	1:100
F4/80	BioLegend	123112	1:20
F4/80	BioLegend	123118	1:100
Foxp3	BioLegend	126419	1:40
FoxP3	Invitrogen	17-5773-82	1:20
H-2Kb	BD Optibuild	742861	1:100
H-2Kb	BD Optibuild	748670	1:20
H-2Kb/H-2Db	BioLegend	114620	1:100
I-A/I-E	BioLegend	107621	1:100
I-A/I-E	Biolegend	107627	1:100
Ki67	BD Horizon	564071	1:20
Ki67	BioLegend	652426	1:200
KLRG1	Thermo	35-5893-82	1:100
Lag3	BioLegend	125227	1:100
Ly6G	BioLegend	127641	1:100
Ly6G	BD Horizon	562700	1:100
Ly6G	BioLegend	127641	1:100
Ly6C	BioLegend	128030	1:100
PD-1	BioLegend	135208	1:100
PD-1	BioLegend	135231	1:100
PD-L1	BioLegend	124315	1:20
PD-L1	BioLegend	124331	1:20
Tbet	BioLegend	644816	1:100
Tim3	eBioscience	11-5870-82	1:100
xCR1	BioLegend	148220	1:100
Zombie UV Fixable Viability	BioLegend	423108	1:100
LIVE/DEAD Fixable Aqua	Invitrogen	L34966	1:600
PerCP-Cy5.5 Rat IgG2b k	BD Biosciences	550764	1:200
APC Armenian Hamster IgG	BioLegend	400912	1:100
AF700 Rat IgG2b k	BioLegend	400628	1:100
APC-Cy7 Rat IgG2a k	BioLegend	400523	1:100
BUV661 Rat IgG2a k	BD Biosciences	612973	1:100
BV605 Rat IgG1 k	BioLegend	400433	1:200
BV650 Rat IgG2a k	BioLegend	400541	1:100
PE Rat IgG2b k	BioLegend	400607	1:100
PE-CF594 Rat IgG2a k	BioLegend	400558	1:100
Pe-Cy7 Rat IgG2a k	BioLegend	400521	1:100

Supplemental Table 2

Western Antibodies			
**Target**	**Company**	**Catalog Number**	**Dilution**
**Phospho-Smad3**	**Abcam**	**ab52903**	**1:250**
**Beta-Actin (N-term) Rabbit**	**Cayman**	**32127**	**1:5000**
**Horseradish Peroxidase-Conjugated Goat Anti-Rabbit IgG**	**Cayman**	**10004301**	**1:4000**
**ECL Western Blotting Detection Reagents**	**Amersham**	**RPN2209**	

Supplemental Table 3
