# Supplementary figures and images for: Pivotal roles for cancer cell–intrinsic mPGES-1 and autocrine EP4 signaling in suppressing antitumor immunity
Source: JCI Insight. 2024 Nov 8;9(21):e178644. doi: 10.1172/jci.insight.178644 (PMC11601572; doi:10.1172/jci.insight.178644)

Supplemental Figure 9B

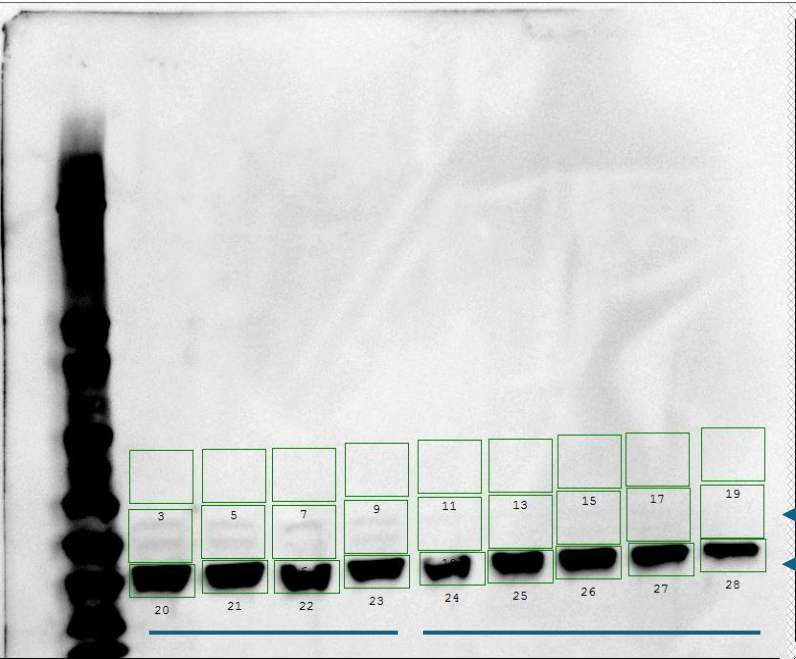

control

Ptger4 KO E10

no treatment

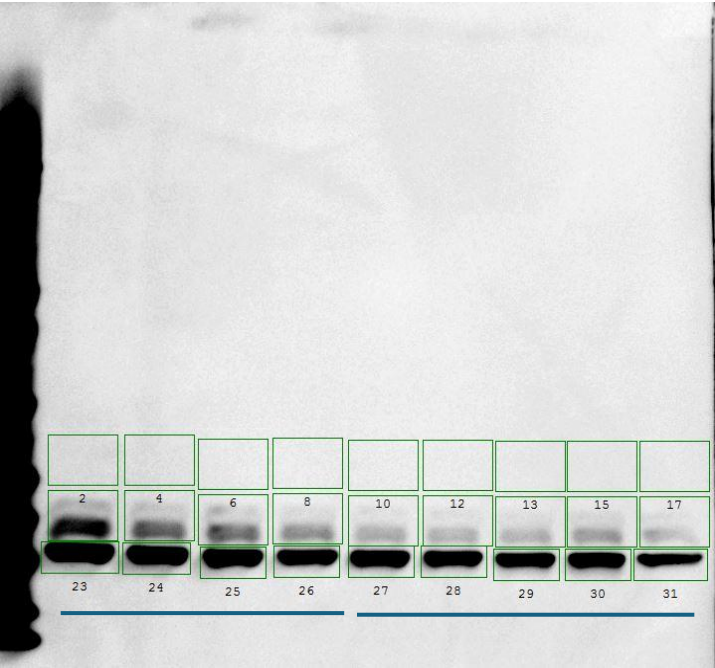

control

Ptger4 KO E10

TGFb+PGE2

Supplement: Unedited blot and gel images [file jciinsight-9-178644-s162.pdf]
